# Synthesis and Antibacterial Properties of Novel Quaternary Ammonium Lignins

**DOI:** 10.1021/acsomega.4c06000

**Published:** 2024-09-02

**Authors:** Mahendra K. Mohan, Harleen Kaur, Merilin Rosenberg, Ella Duvanova, Tiit Lukk, Angela Ivask, Yevgen Karpichev

**Affiliations:** † Department of Chemistry and Biotechnology, Tallinn University of Technology (TalTech), 15 Akadeemia Road, 12618 Tallinn, Estonia; ‡ Institute of Molecular and Cell Biology, University of Tartu, 23 Riia Street, 51010 Tartu, Estonia; § Vasyl’ Stus Donetsk National University, 21 600-richchia Vul., 21027 Vinnytsia, Ukraine

## Abstract

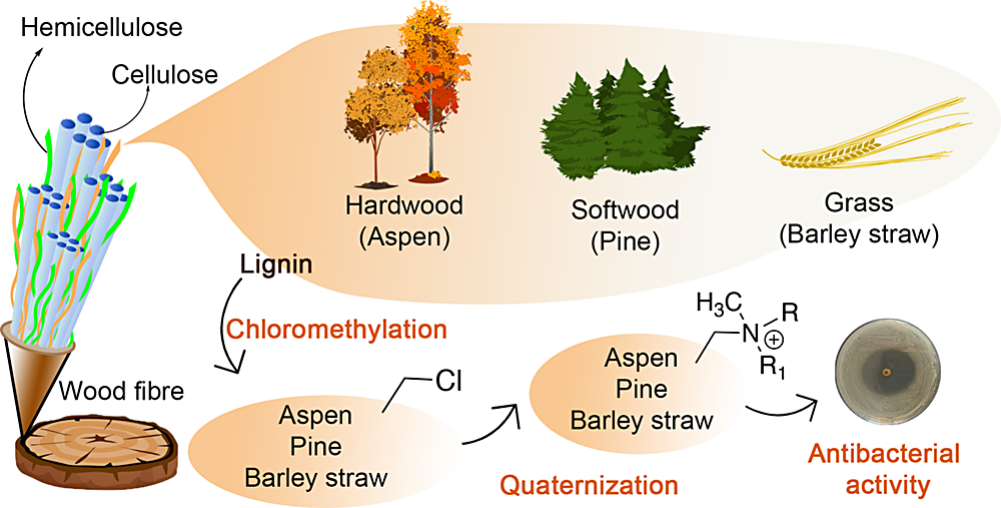

The ongoing demand for effective antimicrobial materials persists, and lignin emerges as a promising natural antibacterial material with renewable properties. The adaptability of lignin to various chemical modifications offers avenues to enhance its antimicrobial activity. Here, we employed chloromethylation and subsequent functionalization with variable tertiary *N-*alkyl dimethyl amines to produce C6–C18 quaternary ammonium lignins (QALs) from hardwood (aspen), softwood (pine), and grass (barley straw). Successful synthesis of QALs was confirmed through NMR and FTIR analysis results along with an increase in the surface ζ-potential. Antibacterial activity of QALs against clinical strains of *Klebsiella pneumoniae* and methicillin-resistant *Staphylococcus aureus* was assessed using minimal bactericidal concentration (MBC) assay and agar growth inhibition zone (ZOI) test. The antibacterial activity of QALs was found to be higher than that of the unmodified lignins. QALs with longer alkyl chains demonstrated an MBC of 0.012 mg/L against *K. pneumoniae* already after 1 h of exposure with similar effect size reached after 24 h for *S. aureus*. For all the lignins, an increase in alkyl chain length resulted in an increase in their bactericidal activity. MBC values of C14–C18 QALs were consistently lower than the MBC values of QALs with shorter alkyl chains. Besides the alkyl chain length, MBC values of barley and pine QALs were negatively correlated with the surface ζ-potential. While alkyl chain length was one of the key properties affecting the MBC values in a liquid-based test, the agar-based ZOI test demonstrated an antibacterial optimum of QALs at C12–C14, likely due to limited diffusion of QALs with longer alkyl chains in a semisolid medium.

## 1 Introduction

Biorenewable polymers characterized by high biocompatibility, biodegradability, and cost-effectiveness have emerged as compelling alternatives for a diverse range of applications.^1^ Among the various sources of renewable carbon, lignocellulosic biomass has gained prominence, with lignin being the most abundant polyphenolic resource.^2^ Lignins have various advantages because of their biocompatibility, antioxidant properties, protection against ultraviolet radiation, and antibacterial activity.^3^

Lignocellulosic biomass is typically composed of 10–40% lignin, with its composition and structure varying depending on the source of lignocellulose.^4^ Postextraction, lignin undergoes several chemical transformations that encompass depolymerization, creation of chemically active sites, chemical alteration of hydroxyl groups, and the generation of lignin graft copolymers.^3^ The final lignin is a three-dimensional network structure, composed of coniferyl alcohol, *p*-hydroxyphenyl, and syringyl groups interconnected by diverse ester and C–C bonds.^5^ Around 70 Mt of lignin is produced by the pulp and paper industry annually, but alarmingly, a mere 5% of it is harnessed into high value added products, such as polymer reinforcement,^6−9^ stabilizers in emulsions, colloidal suspensions,^10,11^ and concrete plasticizers.^12,13^ Approximately 95% of lignin residues are either employed as fuel or discarded directly as liquid waste. Considering the wide variety of application areas of lignin, such practices not only deplete valuable raw material resources but also contribute substantially to environmental pollution.^2,14^ However, recent years have witnessed a growing interest toward exploring lignin’s applications across diverse fields including biomedicine,^15,16^ food packaging,^17−19^ cosmetics, health products,^20,21^ precursor materials for advanced 3D printing,^22^ and antimicrobial applications.^23−25^ The observation on lignin’s antimicrobial properties is not surprising, as plants have evolved to harness these properties as a defense mechanism against invading pathogenic microbes.^26^ Lignin has not only demonstrated its inhibitory effect against plant pathogens *Pseudomonas putida* and *Xanthomonas* sp.^27−29^ but also against other bacteria, e.g., those colonizing plastic surfaces.^30^ In general, the mechanism of lignin’s antibacterial activity is proposed to rely on its strong affinity for bacterial cell surface and interaction with surface proteins and lipids, leading to membrane disruption and inhibition of the respiratory chain.^31^ The described events are expected to result in the formation of reactive oxygen species (ROS), causing oxidative damage to cellular components and hindering the bacterial growth.^29^ In a number of studies, lignins have been further combined with other antimicrobial materials to enhance their antimicrobial effect. Silver nanoparticles have been added to lignins to secure their activity against *Escherichia coli*, *S. aureus*, *Pseudomonas aeruginosa*, *Bacillus subtilis*, and *K. pneumoniae*.^32,33^ It has been proposed that incorporation of silver to lignin and further coating with cationic polyelectrolyte layer eases the interaction between lignin and bacterial membrane and leads to a synergistic antimicrobial effect.^34^

In order to increase the cationic charge density of lignins and their bacteriostatic and bactericidal activity, some of the recent studies have incorporated quaternary ammonium groups to the lignin structure.^35^ Quaternary ammonium compounds (QAC) are cationic surfactants that belong to a group of the most widely used antimicrobials,^36^ which exhibit significant antimicrobial activity due to disruption of bacterial cell membrane and leakage of cellular components.^37−40^ This mechanism occurs due to the presence of a positively charged nitrogen atom connected with four alkyl or aryl groups,^41^ among which one stands out as a lengthy hydrocarbon chain usually with eight or more carbon atoms, therefore acting as a hydrophobic component. It has been clearly shown that the properties of this hydrophobic side chain play a significant role in influencing the antimicrobial properties of QAC.^42^ In general, due to the improved penetration capability of longer alkyl chains to bacterial membranes,^39,43^ the tendency shows that the longer hydrophobic carbon chain length, the higher antimicrobial activity.^44^ However, some studies have demonstrated an optimal side chain length for the best antimicrobial performance of QACs being 10–12 carbons against Gram-negative^45^ and 13–14 carbons against Gram-positive bacteria.^46^ Other studies have shown that this tendency persists in case of surface-active ionic liquids, i.e., QAC with a structure altered by an insertion of an amino acid moiety between quaternary ammonium head group and the side chain.^47,48^ Extension of the alkyl chain length often leads to leveling off or fading of antimicrobial activity, due to the so-called cutoff effect^49^ caused by limited aqueous solubility, kinetic effects, or interactions with biological molecules. In general, Gram-positive bacteria are expected to be more sensitive to QACs than Gram-negative bacteria as the prior lack the outer membrane that restricts QACs access to their target site in cytoplasmic membrane.^50^ Dimeric QACs bearing two cationic groups and two hydrophobic carbon chains have been shown to exhibit greater antibacterial and biocidal activity compared to their monomeric counterparts.^51,52^ Lignins modified with quaternary ammonium groups have been shown to exhibit substantially higher antibacterial activity than unmodified lignins against *E. coli*, *Listeria monocytogenes*, *Salmonella enterica*, and *S. aureus*.^53,54^ Moreover, while QACs have been shown to exhibit notable toxicity to environmental organisms^55^ and eukaryotic cells in vitro,^56^ QAC-modified lignins have been shown to be less cytotoxic.^57^ Therefore, quaternary ammonium lignins (QALs) can be considered as safer analogs to low molecular weight QACs for human use and potentially also from an environmental perspective.

The main strategy of synthesis of QALs involves Mannich amination combined with attachment of the quaternary alkyltrimethyl or alkyltriethylammonium group to the OH groups.^53,57,58^ Recently, our group has presented a greener approach for QAL synthesis that uses chloromethylation step carried out under mild reaction conditions with no Lewis acid catalyst followed by the reaction with a corresponding amine.^59^ In this study, we employed the latter synthesis strategy to design a series of QALs based on three different lignin materials originating from hardwood (aspen), softwood (pine), and grass (barley straw). Quaternary ammonium groups added to those lignins varied between 6 and 18 carbons in their alkyl chain length. The resulting QALs were tested for their physicochemical properties and antibacterial activity against clinical isolates of *S. aureus* and *K. pneumoniae*.

## 2 Results and Discussion

### 2.1 Characterization of Lignins

Considering the constituent units of lignin monomers and their structural composition, lignin is identified as a significantly branched irregular polymer featuring diverse functional groups such as aliphatic and phenolic hydroxyls, carboxylic, carbonyl, and methoxyl groups.^60−62^ In this study, biomasses from three different sources known to have different monolignol compositions and different constituent units were used. The softwood (pine) lignin is primarily (>95%) composed of guaiacyl (G) units, with very minimal contribution (<5%) of hydroxyphenyl (H) units.^63^ The hardwood (aspen) lignin exhibits a more balanced distribution, with H units ranging between 0 and 8%, G units between 25 and 50%, and syringyl (S) units between 45 and 74%. Grass (barley) lignin, on the other hand, displays a wider variability, with H ranging between 5 and 35%, G between 35 and 80%, and S between 20 and 55%.^63^

**Scheme 1:**
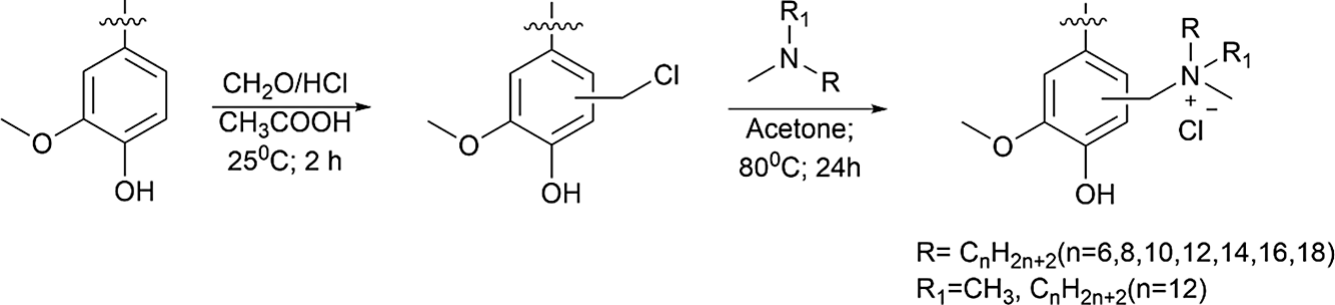
Schematic Synthesis Pathway of Quaternary Ammonium Lignins, as Shown for G Unit of Lignin

**Figure 1:**
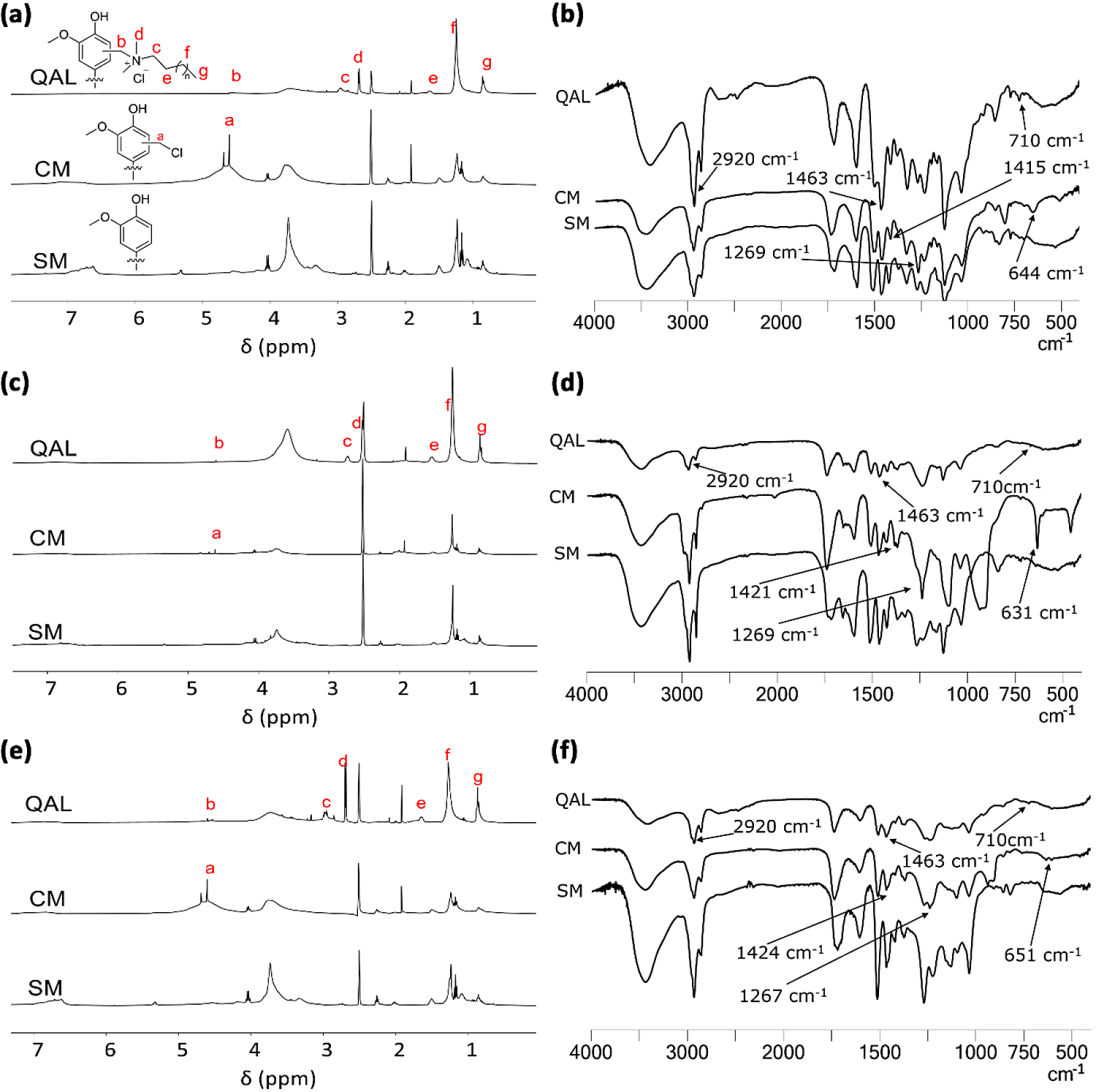
^1^H NMR and FTIR spectra of starting materials (SM), chloromethylated lignins, and quaternary ammonium lignins (as illustrated by the example of C12). ^1^H NMR (a, d, e) and FTIR (b, d, f) spectra of aspen (a, b), barley (c, d), and pine (e, f) lignins. Designation on ^1^H NMR spectra corresponds to the sites highlighted on the molecular formulas drawn in panel (a). In FTIR spectra, characteristic peaks are indicated with arrows.

The richness of chemical sites within lignin has the potential for chemical modifications. In this study, chloromethylation and further quaternization of the chlorinated sites (Scheme 1) were used. Previous studies have demonstrated the successful integration of chloromethylation into aspen lignin,^59^ and the same methodology was used in this study for aspen, barley, and pine lignins. The analysis of ^1^H NMR spectra of original organosolv (SM) and chloromethylated (CM) lignins, as depicted in Figure 1 unequivocally, verifies the chloromethylation process. A distinct new peak (peak (a) in Figure 1) emerges consistently across all three lignin samples, registering at 4.5–4.75 ppm. This peak corresponds specifically to the presence of −CH_2_–Cl within the benzene ring, offering compelling evidence of the successful chloromethylation process in all tested lignin variants. Similarly, distinctive peaks in FTIR at 633–670 cm^–1^ are indicative of −CH_2_–Cl groups (Figure 1). However, the peak around 1413–1424 cm^–1^ can be attributed to aromatic ring vibrations or C–H deformation vibrations in the methylene (−CH_2_−) groups, which are already part of the lignin structure. This means that even without chloromethylation, lignin itself shows an absorption peak in this region, making it less distinctive for identifying the incorporation of −CH_2_–Cl groups specifically. Chloromethylation introduces −CH_2_–Cl groups into the lignin, which should theoretically give rise to new or enhanced peaks in the FTIR spectrum. However, if the existing lignin structure already has vibrations in the same region (1413–1424 cm^–1^), the addition of −CH_2_–Cl groups might not result in a completely new peak but rather a subtle shift or increase in intensity, which could be difficult to distinguish. In some cases (Figure 1b,d,f), a slight shift is observed, suggesting that these peaks might be overlapping. Similarly, the peak at 1264–1267 cm^–1^ is typically associated with C–O stretching in ether groups or possibly with C–Cl stretching. Since lignin has abundant ether linkages, the overlap with the new C–Cl bonds formed during chloromethylation might result in only a slight shift or broadening of the peak rather than a distinct new peak. The similarity between lignin and chloromethylated lignin in this region could be due to the fact that the chloromethyl groups do not significantly alter the existing vibrational characteristics of ether linkages. The extent of chloromethylation and the distribution of −CH_2_–Cl groups within the lignin matrix may also affect the FTIR spectrum. Considering the lignin structure, if the chloromethylation is not uniform or if the concentration of −CH_2_–Cl groups is low, the changes in the FTIR spectrum might be subtle. This could explain why the differences in the range of 1264–1267 cm^–1^ are not pronounced. These findings provide compelling evidence of the successful incorporation of chloromethane into the lignin structure. However, chloromethyl substitutions in the different lignins were different. XRF analysis shows that CM variants of aspen, barley, and pine lignins were functionalized with 20.0, 10.5, and 7.7% chloromethane, respectively. According to earlier studies, chloromethylation may be significantly affected by the monolignol composition of lignins and the presence of hydroxyphenyl (H) units and guaiacyl (G) units, which generally indicate a greater potential for chemical reactivity and functionalization. Our findings show that pine exhibits a relatively restricted distribution of these active sites, whereas aspen and barley offer more diverse monolignol structures, making them potentially more versatile for chloromethylation.

Addition of tertiary dimethyl amines to CM lignins resulted in the formation of QALs as proven by ^1^H NMR and FTIR spectra (Figure 1). ^1^H NMR spectrum demonstrated the “g” and “f” peaks at δ 0.88 and δ 1.25 ppm, indicating the presence of CH_3_ and CH_2_ moieties constituting hydrophobic tails of the alkyl chains. The singlet peak “d” at δ 2.69 ppm indicated the presence of CH_3_ groups connected to quaternary nitrogen. “e” and “c” peaks at δ 1.64 and 2.96 ppm were attributed to the presence of CH_2_ groups next to the quaternary nitrogen. Singlet “b” peaks at δ 4.66 ppm indicated the presence of CH_2_ groups connecting the quaternary nitrogen [ph–(CH_2_)–N]. These findings from NMR spectra aligned well with FTIR, which revealed the appearance of the characteristic absorption band of −(CH_2_)– groups at 710 cm^–1^ in the case of QALs (Figure 1). Moreover, the characteristic absorption bands of CH_3_ and CH_2_ were observed at 2920 and 1463 cm^–1^ regions in the FTIR spectra of all QALs (Figures 1 and S2). Additionally, FTIR spectra of QALs retained most of the characteristics of their SM predecessors, supporting the assertion that the structural integrity of lignin remained largely undisturbed throughout the modification process.

**Figure 2:**
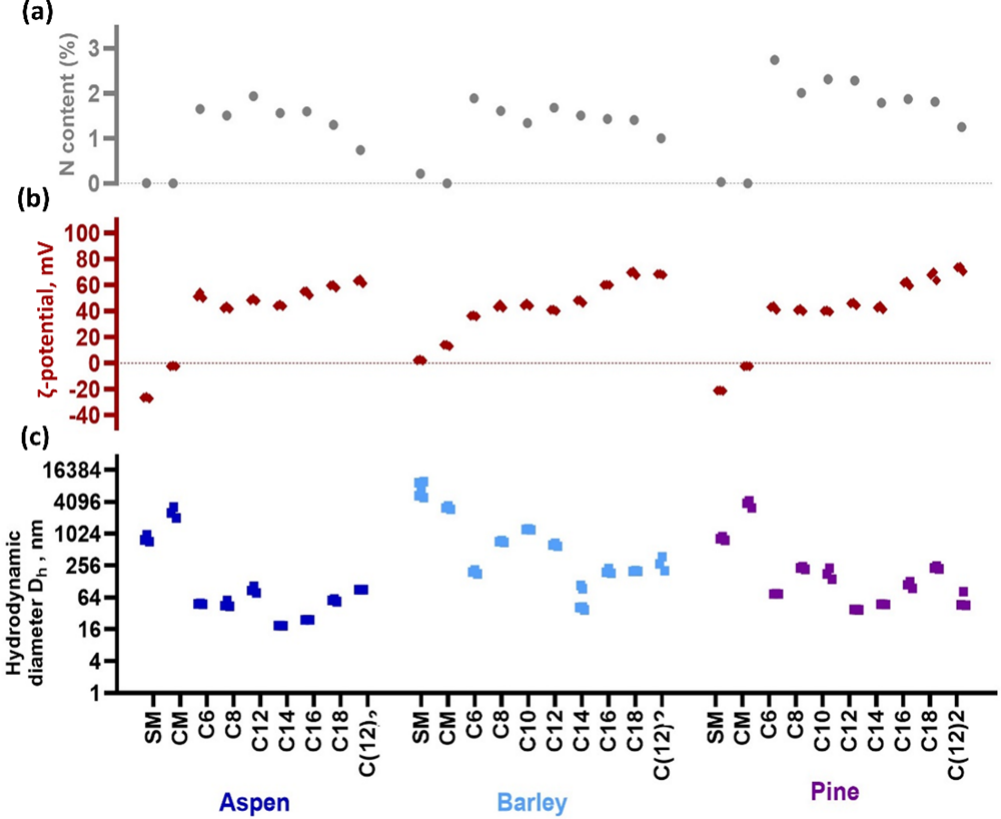
Properties of the lignins and quaternary ammonium lignins (QALs) potentially affect antibacterial activity in minimal biocidal concentration test conditions (1.5% DMSO in water). (a) N content (%) of the samples as a proxy of active moiety content in QALs, (b) hydrodynamic diameter (nm), and (c) ζ-potential (mV). Dotted red and gray lines for ζ-potential and N content represent 0 values.

An additional proof of the successful incorporation of tertiary amines is the appearance of nitrogen in lignins after quaternization (Figure 2, Table S1). Compared with pine and aspen lignin, barley straws contained some nitrogen before the addition of tertiary dimethyl amines. This nitrogen could be attributed to the natural composition of barley material, e.g., the presence of amino acids, and other nitrogen-containing compounds that were retained during lignin extraction process.^64^ The nitrogen content in QALs varied between 0.77 and 2.74% and was dependent on the source of lignin. The highest nitrogen content was detected in pine QALs followed by barley and aspen QALs (Table S1). Considering that one quaternary ammonium group reacted with one chloromethyl group, the amount of chlorine could be used as a predictor for quaternization. However, the finding that pine lignin contained the highest amount of nitrogen and the lowest amount of chlorine (XRF data to determine the content of organic chlorine were discussed above) contradicts the idea of a straightforward relationship. The discrepancy is most likely due to the incomplete reaction of alkyl chains with chloromethyl groups. One likely cause for this could be the solubility of lignin in quaternization reactions affecting the accessibility of reactive chloromethyl sites by the tertiary dimethyl amines, especially those with longer carbon chain lengths. Our earlier observations indicated the superior solubility of pine lignin compared with the other two lignins. This may explain the higher concentration of nitrogen in pine lignin. Furthermore, the structure of aspen lignin has been shown to be relatively inflexible, likely due to the higher number of methyl groups in its monolignols, leading to increased steric hindrance and, consequently, reduced reactivity.^53^ When the N content and carbon chain length of QALs of different lignins was correlated, a significant negative correlation (*r* = −0.80; Table S1) was found for pine lignin, suggesting similar incorporation efficacy of alkyl chain lengths with different number of C atoms. The fact that no statistically significant correlation between N content and C chain length was found for barley and aspen lignins (Table S1 and comparison can also be seen in Figure 2) suggests nonlinearity of quaternization reaction in these lignins in case of different ternary dimethyl amines. When comparing the nitrogen content of C12 and (C12)_2_ QALs, the N content was consistently lower in the case of double chains for all lignins. The reason for the lower incorporation of double chains was likely the steric hindrance. Since the nitrogen content reported in this work is provided specifically for the isolated products, we also suppose that among the factors contributing to the variation in nitrogen content for the different QALs could be some dissolved lignin lost during the isolation step.

The incorporation of quaternary ammonium groups into lignins was also evaluated by ζ potential (mV). Expectedly, the QALs exhibited a positive surface charge, while lignins without quaternary ammonium chain modification exhibited slightly negative, positive, or close to neutral surface charge (Table S1 and Figure 2). For most QALs, the ζ-potential values were higher than + 40 mV, and in the case of barley and pine, the surface charge was also significantly positively correlated with alkyl chain length (Pearson *r* = 0.89 and 0.83, respectively) (Table S1). In case of aspen lignin, the charge did not show significant correlation with alkyl chain length.

As some of the lignin samples were visually aggregated, hydrodynamic particle size of all lignins was analyzed in order to evaluate the stability of their suspensions. These particle size measurements showed that CM and SM without a quaternary ammonium group aggregated significantly in 1.5% DMSO (the final antibacterial test environment), while the particle sizes of QALs were significantly smaller. Therefore, clearly, the high positive surface charge of QALs allowed better dispersion of lignins than the mild positive or negative or close to neutral surface charge of SM and CM. There was no significant correlation between hydrodynamic diameter and alkyl chain length of QALs (Table S1).

### 2.2 Antibacterial Activity of Lignins

**Figure 3:**
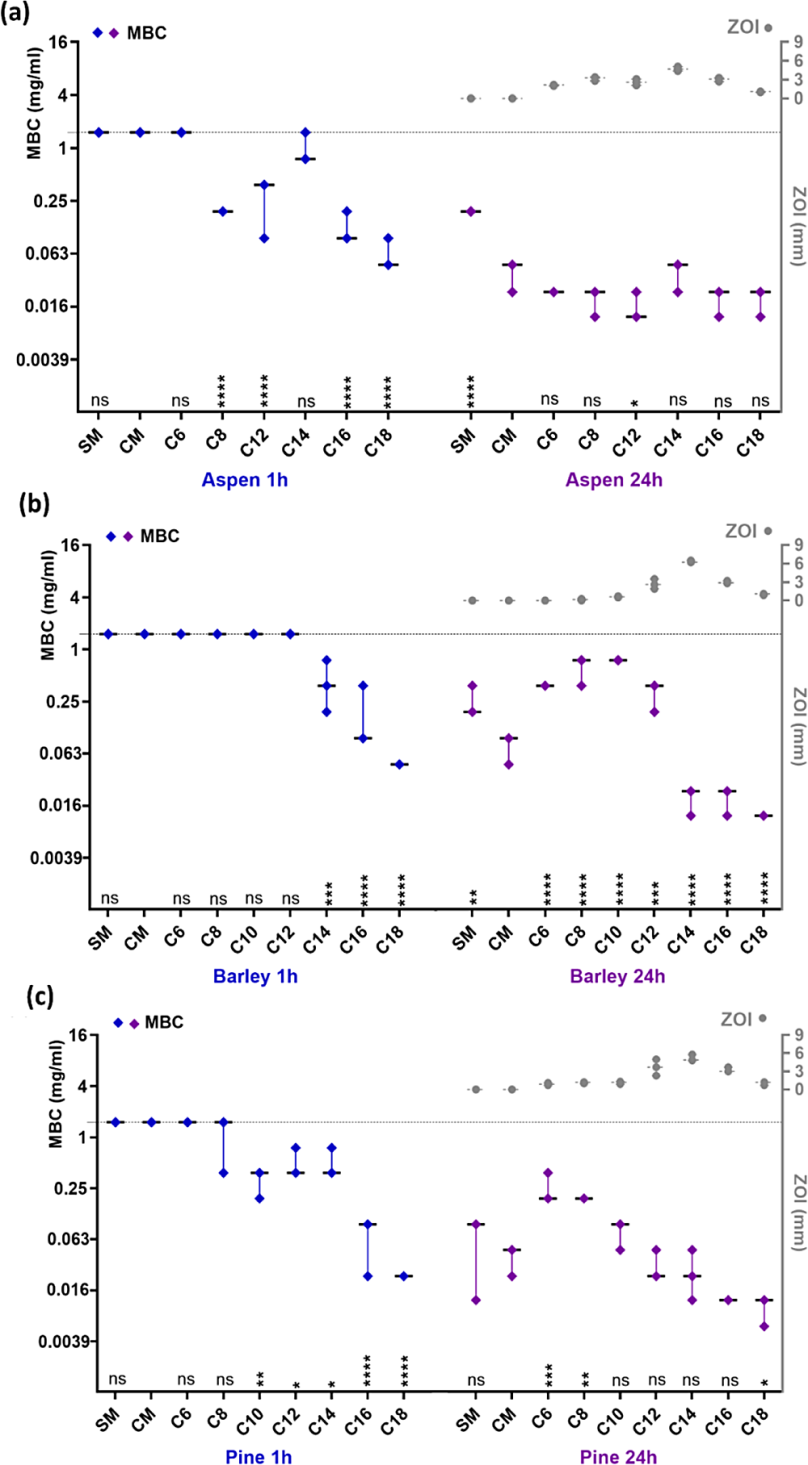
Minimal bactericidal concentration (MBC) of lignin sample against *S. aureus*. MBC values for (a) aspen, (b) barley, and (c) pine after 1 and 24 h of exposure are plotted on the left *Y*-axis, and zone of inhibition values for 24 h of incubation period are on right *Y*-axis. SM – organosolv lignin, CM – chloromethylated lignin, C6–C18–quaternary ammonium lignins, QAL. Median and range of three biological replicates are shown. Highest concentration (1.5 mg/mL) used in MBC assay is shown as a gray dotted line on the left *Y*-axis. Statistically significant differences from control (CM) are presented above *X*-axis for each respective alkyl chain length (C) denoted by ns (not significant), ****(*p* ≤ 0.0001), ***(*p* ≤ 0.001), **(*p* ≤ 0.01), and *(*p* < 0.05).

**Figure 4:**
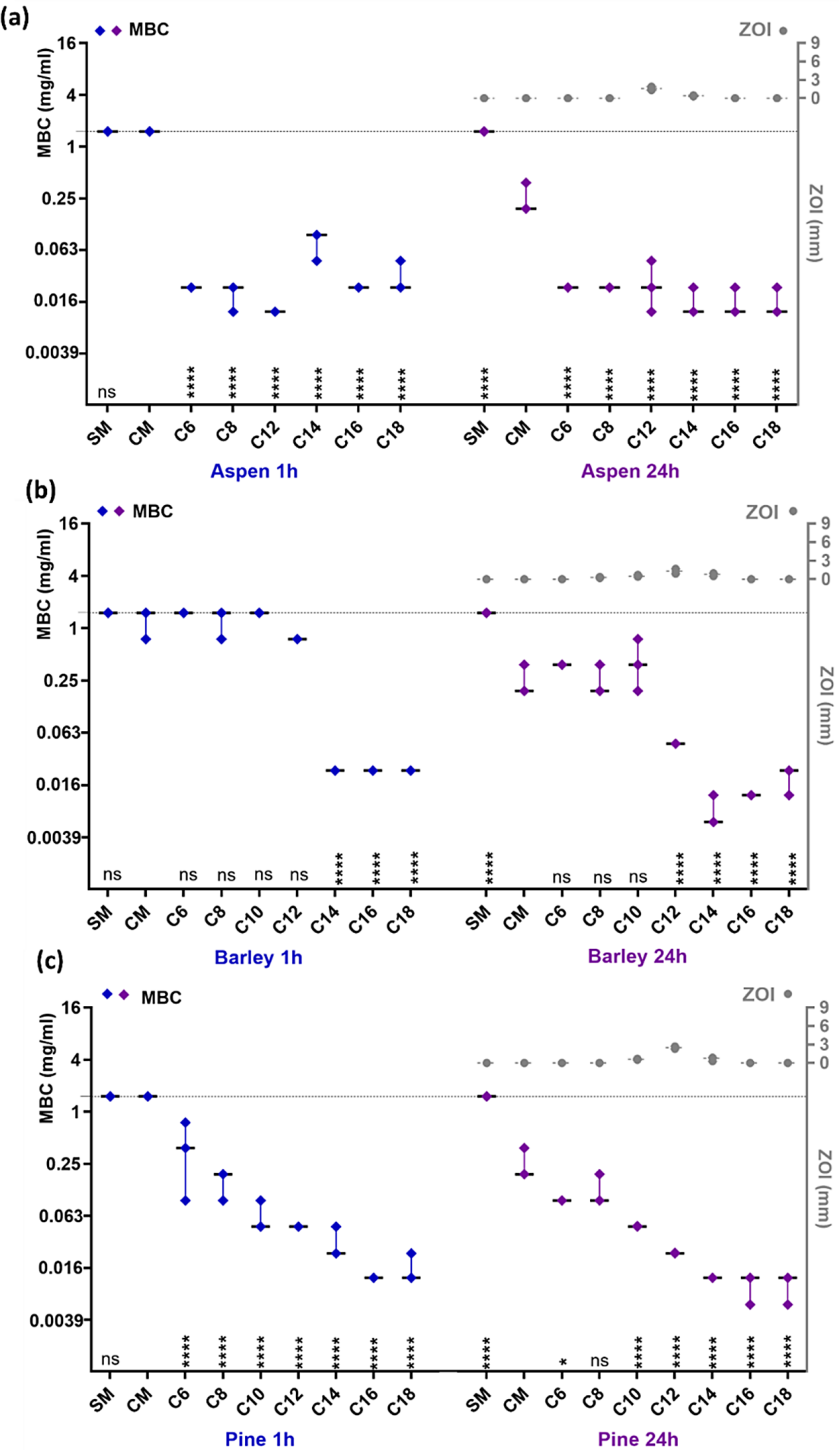
Minimal bactericidal concentration (MBC) of lignin samples against *K. pneumoniae*. MBC values for (a) aspen, (b) barley, and (c) pine after 1 and 24 h of exposure are plotted on the left *Y*-axis, and zone of inhibition values for 24 h of incubation period are on right *Y*-axis. SM – organosolv lignin, CM – chloromethylated lignin, C6–C18–quaternary ammonium lignins, QAL. Highest concentration (1.5 mg/ml) used in MBC assay is shown as a gray dotted line on the left *Y*-axis. Median and range of three biological replicates are shown. Statistically significant differences from control (CM) are presented above *X*-axis for each respective alkyl chain length (C) denoted by ns (not significant), ****(*p* ≤ 0.0001), ***(*p* ≤ 0.001), **(*p* ≤ 0.01), and *(*p* < 0.05)

Antibacterial activity of SM, CM, and QALs was evaluated by measuring their bactericidal (minimal biocidal concentration, MBC) effect in water and bacteriostatic (growth inhibition, ZOI) effect in a semisolid (agar) medium. For both tests, aggregation behavior of the QALs and intrinsic antibacterial activity of their solvent DMSO determined the types of tests and maximum QAL concentrations that could be tested. Pilot experiments showed that while DMSO toxicity was not limiting the ZOI assay, then in the MBC assay, the highest concentration of DMSO that could be tested with Gram-negative *K. pneumoniae* and Gram-positive *S. aureus* was 1.5% (Figures S1 and S3) inherently also limiting the upper concentration limit of QALs (Figures 3 and 4).

Maximum bactericidal effect inflicted by the QALs (MBC of about 0.012 mg/L after 24 h exposure) was similar across samples from all lignin sources (aspen, barley, and pine) and both bacterial models (*K. pneumoniae*, *S. aureus*). However, there were differences in the bactericidal effect of the controls (SM and CM) as well as in the speed of bactericidal action of the QALs (Figures 3 and 4 and Tables S3 and S4) discussed in more detail below.

Our results agree with the general understanding about higher antibacterial effect of lignins to Gram*-*positive bacterial species as opposed to Gram-negatives.^53,65,66^ Based on MBC values, nonquaternized lignins (SM, CM) from all lignin sources were more bactericidal to *S. aureus* than *K. pneumoniae* after 24 h. Specifically, 8–15 times lower concentrations of SM and 2–4 times lower concentrations of CM were needed to kill *S. aureus* compared to *K. pneumoniae* (Tables S3 and S4). CM was generally 2–8 times more bactericidal than SM across both time points and bacterial species, and its bactericidal activity against *S. aureus* was not significantly enhanced by quaternization (Figure 4 and Table S3). Hydrodynamic size of the lignin aggregates could not directly explain the observed differences in bactericidal efficacy of SM and CM. However, SM of all lignin sources was more negatively charged or less positively charged than CM (Table S1 and Figure 2). A decrease of negative or increase in positive charge could respectively decrease electrostatic repulsion or increase attraction to bacterial cell surface, and/or chloromethylation itself might contribute toward an antibacterial effect.

Contrary to the nonquaternized lignins, QALs were generally more bactericidal towards *K. pneumoniae* (Figure 4) than *S. aureus* (Figure 3) after 1h exposure, but the difference in MBC values mostly disappeared after 24 h exposure. QALs effect toward *K. pneumoniae* was more rapid than toward *S. aureus* with the exposure time variable contributing to overall MBC value variability by 6 and 33%, respectively, based on main effects in multiple linear regression (among exposure time, lignin source, N content, alkyl chain length, hydrodynamic size, and ζ-potential). In comparison, the bactericidal effect of CM substantially increased in time also for *K. pneumoniae*, indicating a different underlying mechanism of action of the CMs lignins compared to QALs. Interestingly, literature reported that quaternary ammonium salts of lignin have been shown to be more effective toward *S. aureus* as opposed to Gram-negatives, e.g., with 3-fold difference in MIC values in liquid test format.^72^ However, the exact effect sizes are challenging to compare because antibacterial activity of lignins seems to be dependent on both the extraction method and their chemical structure.^23,25^

QALs with longer alkyl chains (C14–C18) demonstrated more consistent antimicrobial properties across bacterial species and plant origins compared to those with shorter alkyl chains (Figures 3 and 4). QALs with longest alkyl chains also presented the highest positive charge in exposure conditions (Table S1) that could enhance electrostatic attraction toward negatively charged bacterial cell surface and physically enhance the bactericidal effect. QALs with shorter alkyl chains (C6–C12) were generally less effective except for quick-acting antimicrobial potential of C6–C12 of aspen toward *K. pneumoniae* (Figure 4). Interestingly, after 24 h of exposure to *S. aureus*, C6–C12 of barley and pine showed even lower bactericidal effect compared to CM. The latter is largely explained by comparison to the already quite toxic CM control itself, as explained above.

**Figure 5:**
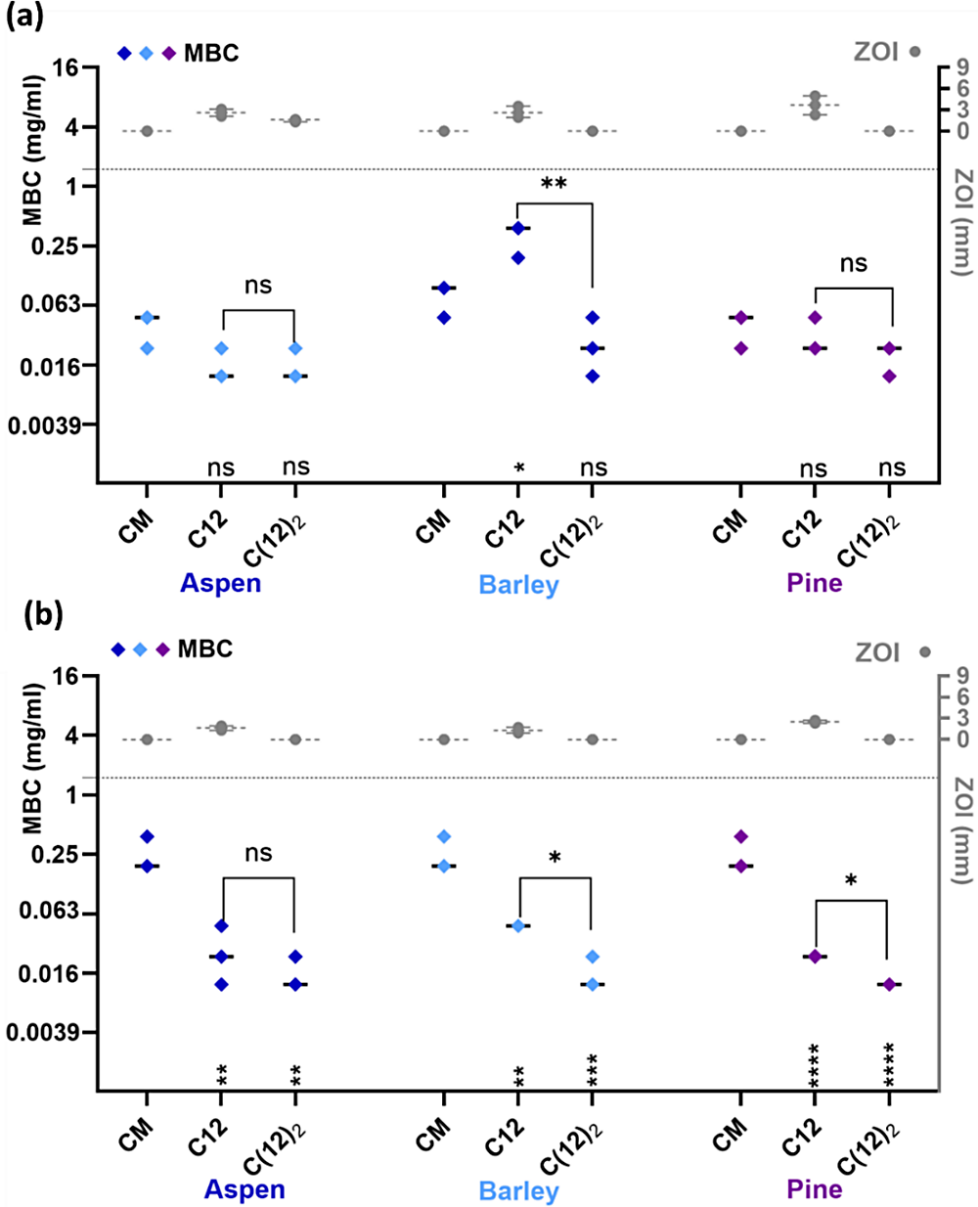
Minimal bactericidal concentration (MBC) of lignin samples modified with either single or double alkyl chains, tested against (a) *S. aureus* and (b) *K. pneumoniae*. MBC values for aspen, barley, and pine after 24 h of exposure are plotted on the left *Y*-axis, and zone of inhibition values for 24 h of incubation period are on right *Y*-axis. CM – chloromethylated lignin, C12, C(12)_2_ – quaternary ammonium lignins, QAL. Median and range of three biological replicates are shown. Highest concentration (1.5 mg/ml) used in MBC assay is shown as a gray dotted line on the left *Y*-axis. Statistical significance of differences (*p* < 0.05) of MBC values from the CM is presented under each respective alkyl chain length (C) and between the single and double chains are marked above (ns(nonsignificant), ****(*p* ≤ 0.0001), ***(*p* ≤ 0.001), **(*p* ≤ 0.01), and *(*p* < 0.05)).

Modifications with a double C12 alkyl moiety instead of a single C12 alkyl chain resulted in inconsistent changes in antibacterial activity (Figure 5). The MBC value of C(12)_2_ of barley and pine decreased for *K. pneumoniae*, in case of *S. aureus*, such a decrease was only observed in case of barley lignin, when compared to C12. In the case of aspen lignin, C12 and C(12)_2_ resulted in similar MBC values for both bacteria. Inconsistencies in MBC values of QALs with single and double alkyl chains can at least partly be explained by the substantial decrease of nitrogen content in the double-chain QALs compared to single-chain QALs, indicating that modification with double alkyl chains was less efficient resulting in the presence of smaller amount of the moieties possessing antibacterial activity at the same QAL concentration. Considering the differences in N content, then based on MBC values, QALs with double C12 appear more toxic to bacteria than QALs with single C12.

MBC values of barley and pine QALs consistently negatively correlated with both alkyl chain length and aggregate charge (Table S1) as QALs with longer alkyl chains and higher ζ-potential proved to be more bactericidal (Figures 3 and 4). The bactericidal effect of aspen QALs did not correlate with either alkyl chain length or aggregate charge, possibly due to quick-acting properties and having reached most of their full potential by the 1 h time point. Additionally, although aspen QALs had the same alkyl chain length modifications, they exhibited a substantially narrower range of ζ potential values across C6–C18 compared to barley and pine QALs. This suggests that aggregate charge and potential electrostatic attraction to negatively charged cell surfaces could enhance the bactericidal activity. This is further illustrated by a strong positive correlation between alkyl chain length and ζ-potential of barley and pine but no significant correlation for aspen QALs (Table S1). Both higher antimicrobial activity at lower alkyl chain lengths and different charge dynamics of aspen QALs could potentially relate to quite different monolignol compositions of aspen lignin compared with pine and barley. It is also not entirely clear what causes the increase of ζ potential with the increase of alkyl chain length of pine and barley QALs. Unfortunately, contributions of possibly causal interactions between the variables in Table 1 to changes in MBC values were not evaluated in multiple linear regression due to intervariable dependencies and multicollinearity.

**Table 1:** Correlations between Bactericidal Effects of Quaternary Ammonium Lignins (Minimal Bactericidal Concentration Values after 24 h Exposure) and Their Physicochemical Properties^a^

	origin of lignin	alkyl chain length	N content	hydrodynamic size	ζ- potential
*K. pneumoniae*	Aspen	–0.39(ns)	0.41(ns)	0.43(ns)	–0.21(ns)
	Barley	–0.72(***)	0.21(ns)	0.55(**)	–0.52(*)
	Pine	–0.80(****)	0.47(*)	0.29(ns)	–0.54(*)
*S. aureus*	Aspen	0.043(ns)	–0.15(ns)	–0.52(*)	–0.31(ns)
	Barley	–0.75(****)	0.11(ns)	0.85(****)	–0.61(**)
	Pine	–0.84(****)	0.66(**)	0.08(ns)	–0.52(*)

a Pearson correlation coefficients (*r*) and their statistical significance (in brackets) are shown. Statistical significance is ns (not significant), ****(*p* ≤ 0.0001), ***(*p* ≤ 0.001), **(*p* ≤ 0.01), or *(*p* < 0.05).

In general, most of the earlier studies have indicated that among quaternary ammonium compounds with the most used alkyl chain lengths of C8–C18, there is an optimal chain length usually above 10 carbons that grants the highest antibacterial efficacy depending on the compound, exposure time, and the type of bacterial cell wall.^44,67−71^ At the same time, QACs with alkyl chain lengths <4 or >18 are considered virtually inactive.^72,73^ Our study showed that in MBC assays, QALs with the longest alkyl chains were the most bactericidal, and no distinct shorter optimum was revealed. Most of the earlier studies that indicated the existence of alkyl chain length optimum have based their antibacterial effect assessment on growth inhibition tests on semisolid (agar) medium by measuring ZOI. When a similar assay with QALs was carried out in our study, we were also able to demonstrate an optimal bacteriostatic effect at C12–C14, whereas no growth inhibition by the QALs with longest alkyl chains was observed (Figures 4 and 5). The alkyl chain lengths that deliver the maximum effect in growth inhibition on agar medium (ZOI) and bactericidal assay in liquid environment (MBC) differ substantially. ZOI shows a sharp optimum at C12 or C14 (depending on bacterial species), with a decline toward C18. In contrast, the MBC assay shows an increasing bactericidal effect with longer alkyl chains, peaking at C16–C18. Similar discrepancy between the results of growth inhibition and bactericidal assays of benzalkonium chloride with variable alkyl chain lengths has also been noted by Tomlinson et al.^73^ We suspect that the ZOI optima are due to differences in hydrophobic aggregation and/or limited diffusion of the QALs with longer alkyl chains in agar medium, rather than their intrinsic biological activity per se. Notably, C14 QALs also formed the smallest aggregates in the water suspension (Figure 3). Figure 5 further illustrates that modification with double alkyl chains compared to single alkyl chains causes discrepancy between the ZOI and MBC results. While ZOI always decreased for double chains compared to single chains, MBC of double chains either remained similar to single chains or even decreased. These discrepancies highlight that inhibition zones can only be used and compared based on the presumption of equal diffusion of the substances of interest in the water environment of semisolid agar medium. As our results demonstrate, ZOI-based bacteriostatic properties of QALs with longer alkyl chains or double alkyl chains of hydrophobic nature can be falsely underestimated by diffusion-limited test formats.

## 3 Conclusions

Here, we present the synthesis and antibacterial characterization of quaternary ammonium derivatives of lignin (QAL) sourced from three origins: hardwood represented by aspen, softwood represented by pine, and grass straws represented by barley straw. Lignin was extracted using organosolv methodology, chloromethylated, and subsequently reacted with the corresponding tertiary *n*-alkyl dimethyl amine with alkyl chain lengths ranging from 6 to 18 carbons (C6–C18). Additionally, for C12, double-chain derivatives (C(12)_2_) were prepared by reaction with corresponding dialkyl methylamines. The original organosolv lignin (SM), chloromethylated lignin (CM), and the final QAL products were characterized by ^1^H NMR and FTIR analysis to describe the products, elemental analysis to determine nitrogen content, XRF to determine organic chlorine content, and ζ-potential to assess the surface charge and hydrodynamic diameter. Nitrogen content analysis revealed the highest and most consistent incorporation of quaternary ammonium moieties in pine lignin. Compared with SM and CM, the QALs exhibited higher positive charges, with significant positive correlation observed between ζ-potential and alkyl chain length of the quaternary ammonium group in pine and barley lignin.

Antibacterial effect of lignins was evaluated by MBC and agar growth inhibition test (zone of inhibition, ZOI) against clinical isolates of *K. pneumoniae* and *S. aureus* MRSA. The non-quaternized lignins (SM, CM) showed no bactericidal effect after 1 h; however, some level of bactericidal activity was detected after 24 h of exposure, particularly against *S. aureus*. Compared with SM, CM exhibited a higher bactericidal effect, likely due to its less negative surface charge or the presence of chlorine in the molecule. Incorporation of quaternary ammonium groups into the lignin increased the antibacterial activity. QALs with longer alkyl chains demonstrated the MBC of 0.012 mg/L against *K. pneumoniae* after just 1 h of exposure, achieving a similar effect size against *S. aureus* after 24 h. For both tested bacteria, QALs with longer alkyl chains (C14–C18) demonstrated a higher bactericidal effect as compared to those with shorter alkyl chains. MBC values of barley and pine QALs correlated negatively with both the alkyl chain length and surface ζ-potential of the QAL aggregates. However, no such clear correlations were found for aspen QALs, likely due to their more consistent aggregate surface charge across different alkyl chain lengths. QALs with a double C12 alkyl moiety showed inconsistent changes in antibacterial activity compared to C12 with a single alkyl chain at the same lignin concentration. However, considering large differences in active moiety content (N content) of C12 double and single chains, double C12 appeared more bactericidal than single C12. Contrary to several previous studies demonstrating an optimal alkyl chain length for antibacterial action of QALs, our study showed that in MBC assays, QALs with the longest alkyl chains were the most bactericidal, and no distinct shorter optimum among C6–C18 was revealed. However, a clear optimum at C12–C14 was observed in the growth inhibition test (ZOI), suggesting that in the case of antibacterial tests carried out in agar, the growth inhibiting effect may be restricted by diffusion of QALs with longer alkyl chains along with their bacteriostatic properties. Therefore, semisolid diffusion-limited antibacterial tests should be avoided in the efficacy assessment of compounds with potentially different aggregation and/or diffusion properties in aqueous environments.

Although we demonstrated the incorporation of quaternary ammonium groups with longer alkyl chains into biorenewable lignin material in the development of effective bactericidal materials, concerns have been raised regarding the toxicity of QACs. Therefore, before the actual applications, both the environmental and cytotoxicity of the promising QALs should be investigated.

## 4 Method

### 4.1 Materials

Ethanol, acetonitrile, hexane, hydrochloric acid, acetic acid, sulfuric acid, and DMSO-*d*_6_ were purchased from Sigma-Aldrich (Taufkirchen, Germany). All of the reagents used were of analytical reagent grade. Deionized water from a Milli-Q water purification system (Millipore S.A., Molsheim, France) was used throughout the study. Aspen wood chips were provided by Estonian Cell AS (Kunda, Estonia); longitudinally sawn pine timber sawdust was provided by Prof. Jaan Kers (Tallinn University of Technology, Tallinn, Estonia); and barley straw was provided by Prof. Timo Kikas (Estonian University of Life Sciences, Tartu, Estonia). All feedstocks were dried in a convection oven at 50 °C up to 8% moisture, followed by grinding to a fine powder and stored in plastic bags at room temperature.

### 4.2 Extraction and Modification of Lignins

Lignin was extracted from aspen, pine, and barley straw according to the previously described organosolv procedure.^74,75^ A 50 g sample of ground and dried chips of aspen, sawdust of pine, or barley straws was refluxed in a 2 l round-bottom flask equipped with a mechanical stirrer and a condenser, using 1.5 l of solvent for 6 h. The solvent mixture consisted of 0.28 M HCl (37% purity) in absolute ethanol. Subsequently, the mixture underwent filtration through Whatman filter paper, and the solid residue was removed. The collected filtrate was then concentrated to approximately 100 mL using a rotary evaporator. To recover lignin from the pretreatment solution, a precipitation method was employed. The pretreatment liquor was dissolved in 100 mL of acetone and introduced into a vigorously stirred 2 L volume of cold Milli-Q water, reducing the solubility of lignin. The mixture was stirred for 60 min, followed by the separation of the precipitated lignin via centrifugation at 4200 rpm. The retrieved lignin was washed three times with 1 L of ultrapure water, centrifuged, and subsequently dried in a convection oven at 40 °C for 24 h. The dried organosolv lignin was then weighed (yield 6%) and used for either subsequent analysis or the following procedures. The extracted lignin was designated as **SM** (starting material).

Chloromethylation of organosolv lignin was performed according to the previously described procedure.^60^ 1 g of organosolv lignin and 1 g of paraformaldehyde were dissolved in 10 mL of glacial acetic acid and then bubbled with HCl gas for 2 h after which the reaction was stopped by adding 30 mL of water. The product was then filtered, washed with water, and dried in vacuum. The conversion into chloromethylated products was monitored by organic chlorine content analysis. The resulting chloromethylated lignins were named **CM**.

To prepare QALs, to a solution of a CM lignin (1 g in 20 mL of acetonitrile), 1 g of one of the following ternary dimethyl amines, C_6_H_13_N(CH_3_)_2_ C_8_H_17_N(CH_3_)_2_, C_10_H_21_N(CH_3_)_2_, C_12_H_25_N(CH_3_)_2_, C_14_H_29_N(CH_3_)_2_, C_16_H_33_N(CH_3_)_2_, and C_18_H_37_N(CH_3_)_2_, was added. The mixtures were heated at 80 °C for 24 h after which the resulting QALs were filtered, washed with hexane, and dried in vacuum. The resulting QALs were designated as **C6**, **C8, C10**, **C12**, **C14**, **C16, and C18**, depending on the number of carbons in the alkyl chain. One additional modification of CM lignin was synthesized using ternary double *n*-alkyl chain amine (C_12_H_25_)_2_N(CH_3_). The resulting double alkyl chain QAL was designated as **(C12)**_**2**_.

### 4.3 Characterization of Lignins

Proton nuclear magnetic resonance (^1^H NMR) spectra of SM, CM, and QALs were acquired using Bruker Avance III 400 MHz spectrometer (USA). All of the samples (ca. 60 mg) were dissolved in DMSO-*d*_6_ in a 5 mm NMR tube; MestReNova x64 software was used to plot the ^1^H NMR spectra. Fourier transform infrared spectroscopy (FTIR) spectra of the lignins were collected with the Shimadzu IRTracer-100 spectrometer (Kyoto, Japan). The samples were prepared with KBr pellets at a concentration of 1:100 weight. The resolution was set to 2 cm^–1^ with 80 scans recorded. The data analysis was conducted using Shimadzu Lab Solutions software. Elemental analysis for nitrogen was carried out using an Elementar Vario MICRO cube (Langenselbold, Germany) in CHNS mode. XRF analysis of lignins to determine organic chlorine content was carried out using a Bruker S4 Pioneer XRF spectrometer (USA) using a precalibrated MultiRes measurement method. Lignins were mixed 1:10 with NaHCO_3_ for the measurement. Hydrodynamic diameter (*D*_h_) and ζ-potential of the QALs were measured from 1.5 mg/mL lignin suspension in 1.5% DMSO in water using a Zetasizer Nano ZSP instrument (Malvern Panalytical, Malvern, UK). Three to five measurements with 12–15 runs of measurements for each repetition were performed for each sample depending on the homogeneity of the sample.

### 4.4 Antibacterial Activity Assessment

Antibacterial activity of lignin compounds was determined by two methods, growth inhibition assay (zone of inhibition, ZOI), and MBC assessment, using two clinical isolates from Estonian Electronic Microbial dataBase (https://eemb.ut.ee), *S. aureus* strain HUMB 19594 showing methicillin resistance (MRSA) and *K. pneumoniae* HUMB 01336.^76^ Bacteria were routinely cultivated on LB agar medium (5 g/L yeast extract, 10 g/L tryptone, 5 g/L NaCl, 15 g/L agar) and TSA agar medium (17 g/L pancreatic digest of casein, 3 g/L papaic digest of soybean meal, 2.5 g/L dextrose (glucose), and 2.5 g/L dipotassium hydrogen phosphate (5 g/L sodium chloride, 15 g/L agar). Prior to antibacterial tests, lignin samples were dissolved in DMSO at a concentration of 100 mg/mL.

#### 4.4.1 Growth Inhibition Assay

A single colony was picked from overnight growth plates and inoculated into 5 mL of LB broth, after which it was grown for 16 h at 37 °C and 150 rpm shaking. Then, the bacterial culture was diluted with fresh medium 1:50 and incubated for 2 h to reach the exponential growth phase. OD at 600 nm of the culture was then diluted to a target value of 0.1, and 100 μL of the resulting bacterial inoculum was spread uniformly on TSA agar plates using sterile glass beads. The plates were allowed to dry for 5 min. To the freshly inoculated plates, 3 μL drops of the test compounds at 100 mg/mL in DMSO were pipetted. A drop of 3 μL of DMSO was used as a control. The plates were incubated at 37 °C for 24 h for optimal growth after which a transparent growth inhibition zone (measured in mm) around the droplets of the compounds was measured using a caliper. The test was performed in three biological replicates.

#### 4.4.2 Minimal Bactericidal Concentration (MBC)

A single colony from the LB agar plate was inoculated to LB broth and grown for 16 h at 150 rpm at 37 °C. Then, the bacterial culture was diluted with fresh media 1:50 and cultivated at 37 °C and 150 rpm to reach the exponential growth phase (OD 0.6 at 600 nm). The cells were then centrifuged at 5000 g for 10 min at 4 °C, and the pellet was resuspended in an equal volume of sterile water. The previous washing step was repeated twice, and finally, the pellet was suspended in water to target the desired cell density of OD600 = 0.2. The compounds were diluted to the specified concentrations using 3% DMSO, that was selected according to preexperiments where 1:1 diluted amount of 3% DMSO (final concentration of DMSO 1.5%) had no significant effect on *S. aureus* and *K. pneumoniae* viability after 24 h of exposure (Figure S1). Therefore, the highest tested concentration of lignin in this testing format was 1.5 mg/mL, and the diluent was always 1.5% DMSO in water. 100 μL of the bacterial suspension was mixed with 100 μL of lignin solution and incubated at 37 °C for 24 h. After 1 and 24 h of exposure, 3 μL of the cell suspension was drop-plated onto LB agar medium and incubated at 37 °C for 24 h. The lowest concentration of compounds resulting in no visible viable colony formation on agar medium in the 3 μL spot was defined as MBC. MBC tests were carried out in three biological replicates.

### 4.5 Statistical Analysis

Statistical analysis of the data was performed with GraphPad Prism 10.1.1 (GraphPad Software, San Diego, USA). Correlations, multiple linear regression, and analysis of variance (ANOVA) followed by post hoc testing for multiple comparisons at α = 0.05 were used where appropriate.

## Supporting Information Available

Effect of DMSO on bacterial viability FTIR spectra of quaternary ammonium lignins zone of inhibition in growth inhibition test on agar for DMSO and quaternary ammonium lignins for *S. aureus* and *K. pneumoniae*; nitrogen content, hydrodynamic diameter (*D*_h_), and ζ-potential of lignin samples zone of inhibition (ZOI, mm) for *S. aureus* and *K. pneumoniae* MBC of lignin and QAL samples against *S. aureus*; and MBC of lignin and QAL samples against *K. pneumoniae* (PDF)

## Abbreviations

AcOH: Acetic acid
SM: Starting material
CM: Chloromethylated Lignin
QAL: Quaternary ammonium lignin
CHO: Formaldehyde
HCl: Hydrochloric acid
DMSO: Dimethylsulfoxide
EtOH: Ethanol
FTIR: Fourier Transform Infrared Spectroscopy
NMR: Nuclear Magnetic Resonance
PFA: paraformaldehyde
XRF: X-ray Fluorescence

## References

[ref1] Ramakrishna S.; Mayer J.; Wintermantel E.; Leong K. W. Biomedical Applications of Polymer-Composite Materials: A Review. Compos. Sci. Technol. 2001, 61 (9), 1189–1224. 10.1016/S0266-3538(00)00241-4.

[ref2] Laurichesse S.; Avérous L. Chemical Modification of Lignins: Towards Biobased Polymers. Prog. Polym. Sci. 2014, 39 (7), 1266–1290. 10.1016/j.progpolymsci.2013.11.004.

[ref3] Figueiredo P.; Lintinen K.; Hirvonen J. T.; Kostiainen M. A.; Santos H. A. Properties and Chemical Modifications of Lignin: Towards Lignin-Based Nanomaterials for Biomedical Applications. Prog Mater Sci 2018, 93, 233–269. 10.1016/j.pmatsci.2017.12.001.

[ref4] Upton B. M.; Kasko A. M. Strategies for the Conversion of Lignin to High-Value Polymeric Materials: Review and Perspective. Chem Rev 2016, 116 (4), 2275–2306. 10.1021/acs.chemrev.5b00345. 26654678

[ref5] He X.; Luzi F.; Yang W.; Xiao Z.; Torre L.; Xie Y.; Puglia D. Citric Acid as Green Modifier for Tuned Hydrophilicity of Surface Modified Cellulose and Lignin Nanoparticles. ACS Sustain Chem Eng 2018, 6 (8), 9966–9978. 10.1021/acssuschemeng.8b01202.

[ref6] Fan Q.; Liu T.; Zhang C.; Liu Z.; Zheng W.; Ou R.; Wang Q. Extraordinary Solution-Processability of Lignin in Phenol–Maleic Anhydride and Dielectric Films with Controllable Properties. J Mater Chem A Mater 2019, 7 (40), 23162–23172. 10.1039/C9TA06665A.

[ref7] Liu W.; Yao Y.; Fu O.; Jiang S.; Fang Y.; Wei Y.; Lu X. Lignin-Derived Carbon Nanosheets for High-Capacitance Supercapacitors. RSC Adv. 2017, 7 (77), 48537–48543. 10.1039/C7RA08531A.

[ref8] Zhang X.; Liu W.; Yang D.; Qiu X. Biomimetic Supertough and Strong Biodegradable Polymeric Materials with Improved Thermal Properties and Excellent UV-Blocking Performance. Adv. Funct. Mater. 2019, 29 (4), 1806912 10.1002/adfm.201806912.

[ref9] Liu W.; Fang C.; Wang S.; Huang J.; Qiu X. High-Performance Lignin-Containing Polyurethane Elastomers with Dynamic Covalent Polymer Networks. Macromolecules 2019, 52 (17), 6474–6484. 10.1021/acs.macromol.9b01413.

[ref10] Gharehkhani S.; Ghavidel N.; Fatehi P. Kraft Lignin–Tannic Acid as a Green Stabilizer for Oil/Water Emulsion. ACS Sustain Chem Eng 2019, 7 (2), 2370–2379. 10.1021/acssuschemeng.8b05193.

[ref11] Bai L.; Greca L. G.; Xiang W.; Lehtonen J.; Huan S.; Nugroho R. W. N.; Tardy B. L.; Rojas O. J. Adsorption and Assembly of Cellulosic and Lignin Colloids at Oil/Water Interfaces. Langmuir 2019, 35 (3), 571–588. 10.1021/acs.langmuir.8b01288. 30052451 PMC6344914

[ref12] Schmidt B. V. K. J.; Molinari V.; Esposito D.; Tauer K.; Antonietti M. Lignin-Based Polymeric Surfactants for Emulsion Polymerization. Polymer (Guildf) 2017, 112, 418–426. 10.1016/j.polymer.2017.02.036.

[ref13] Kalliola A.; Vehmas T.; Liitiä T.; Tamminen T. Alkali-O2 Oxidized Lignin–A Bio-Based Concrete Plasticizer. Ind Crops Prod 2015, 74, 150–157. 10.1016/j.indcrop.2015.04.056.

[ref14] Lievonen M.; Valle-Delgado J. J.; Mattinen M.-L.; Hult E.-L.; Lintinen K.; Kostiainen M. A.; Paananen A.; Szilvay G. R.; Setälä H.; Österberg M. A Simple Process for Lignin Nanoparticle Preparation. Green Chemistry 2016, 18 (5), 1416–1422. 10.1039/C5GC01436K.

[ref15] Larrañeta E.; Imízcoz M.; Toh J. X.; Irwin N. J.; Ripolin A.; Perminova A.; Domínguez-Robles J.; Rodríguez A.; Donnelly R. F. Synthesis and Characterization of Lignin Hydrogels for Potential Applications as Drug Eluting Antimicrobial Coatings for Medical Materials. ACS Sustain Chem Eng 2018, 6 (7), 9037–9046. 10.1021/acssuschemeng.8b01371. 30023141 PMC6046221

[ref16] Gan D.; Xing W.; Jiang L.; Fang J.; Zhao C.; Ren F.; Fang L.; Wang K.; Lu X. Plant-Inspired Adhesive and Tough Hydrogel Based on Ag-Lignin Nanoparticles-Triggered Dynamic Redox Catechol Chemistry. Nat Commun 2019, 10 (1), 1487 10.1038/s41467-019-09351-2. 30940814 PMC6445137

[ref17] Shankar S.; Rhim J.-W. Preparation and Characterization of Agar/Lignin/Silver Nanoparticles Composite Films with Ultraviolet Light Barrier and Antibacterial Properties. Food Hydrocoll 2017, 71, 76–84. 10.1016/j.foodhyd.2017.05.002.

[ref18] Yang W.; Owczarek J. S.; Fortunati E.; Kozanecki M.; Mazzaglia A.; Balestra G. M.; Kenny J. M.; Torre L.; Puglia D. Antioxidant and Antibacterial Lignin Nanoparticles in Polyvinyl Alcohol/Chitosan Films for Active Packaging. Ind Crops Prod 2016, 94, 800–811. 10.1016/j.indcrop.2016.09.061.

[ref19] Yang W.; Weng Y.; Puglia D.; Qi G.; Dong W.; Kenny J. M.; Ma P. Poly(Lactic Acid)/Lignin Films with Enhanced Toughness and Anti-Oxidation Performance for Active Food Packaging. Int J Biol Macromol 2020, 144, 102–110. 10.1016/j.ijbiomac.2019.12.085. 31838072

[ref20] Qian Y.; Qiu X.; Zhu S. Lignin: A Nature-Inspired Sun Blocker for Broad-Spectrum Sunscreens. Green Chemistry 2015, 17 (1), 320–324. 10.1039/C4GC01333F.

[ref21] de Araújo Padilha C. E.; da Costa Nogueira C.; Oliveira Filho M. A.; de Santana Souza D. F.; de Oliveira J. A.; dos Santos E. S. Valorization of Cashew Apple Bagasse Using Acetic Acid Pretreatment: Production of Cellulosic Ethanol and Lignin for Their Use as Sunscreen Ingredients. Process Biochemistry 2020, 91, 23–33. 10.1016/j.procbio.2019.11.029.

[ref22] Cusola O.; Rojas O. J.; Roncero M. B. Lignin Particles for Multifunctional Membranes, Antioxidative Microfiltration, Patterning, and 3D Structuring. ACS Appl Mater Interfaces 2019, 11 (48), 45226–45236. 10.1021/acsami.9b16931. 31702895

[ref23] Morena A. G.; Tzanov T. Antibacterial Lignin-Based Nanoparticles and Their Use in Composite Materials. Nanoscale Adv 2022, 4 (21), 4447–4469. 10.1039/D2NA00423B. 36341306 PMC9595106

[ref24] Morena A. G.; Bassegoda A.; Natan M.; Jacobi G.; Banin E.; Tzanov T. Antibacterial Properties and Mechanisms of Action of Sonoenzymatically Synthesized Lignin-Based Nanoparticles. ACS Appl Mater Interfaces 2022, 14 (33), 37270–37279. 10.1021/acsami.2c05443. 35960019 PMC9412960

[ref25] Li K.; Zhong W.; Li P.; Ren J.; Jiang K.; Wu W. Antibacterial Mechanism of Lignin and Lignin-Based Antimicrobial Materials in Different Fields. Int J Biol Macromol 2023, 252, 126281 10.1016/j.ijbiomac.2023.126281. 37572815

[ref26] Bhuiyan N. H.; Selvaraj G.; Wei Y.; King J. Role of Lignification in Plant Defense. Plant Signal Behav 2009, 4 (2), 158–159. 10.4161/psb.4.2.7688. 19649200 PMC2637510

[ref27] Espinoza-Acosta J. L.; Torres-Chávez P. I.; Ramírez-Wong B.; López-Saiz C. M.; Montaño-Leyva B. Antioxidant, Antimicrobial, and Antimutagenic Properties of Technical Lignins and Their Applications. Bioresources 2016, 11 (2), 5452–5481. 10.15376/biores.11.2.Espinoza_Acosta.

[ref28] Alves M. J.; Ferreira I. C. F. R.; Froufe H. J. C.; Abreu R. M. V.; Martins A.; Pintado M. Antimicrobial Activity of Phenolic Compounds Identified in Wild Mushrooms, SAR Analysis and Docking Studies. J. Appl. Microbiol. 2013, 115 (2), 346–357. 10.1111/jam.12196. 23510516

[ref29] Yang W.; Fortunati E.; Gao D.; Balestra G. M.; Giovanale G.; He X.; Torre L.; Kenny J. M.; Puglia D. Valorization of Acid Isolated High Yield Lignin Nanoparticles as Innovative Antioxidant/Antimicrobial Organic Materials. ACS Sustain Chem Eng 2018, 6 (3), 3502–3514. 10.1021/acssuschemeng.7b03782.

[ref30] He T.; Jiang Y.; Chang S.; Zhou X.; Ji Y.; Fang X.; Zhang Y. Antibacterial and High-Performance Bioplastics Derived from Biodegradable PBST and Lignin. Ind Crops Prod 2023, 191, 115930 10.1016/j.indcrop.2022.115930.

[ref31] Alzagameem A.; Klein S. E.; Bergs M.; Do X. T.; Korte I.; Dohlen S.; Hüwe C.; Kreyenschmidt J.; Kamm B.; Larkins M.; Schulze M. Antimicrobial Activity of Lignin and Lignin-Derived Cellulose and Chitosan Composites against Selected Pathogenic and Spoilage Microorganisms. Polymers (Basel) 2019, 11 (4), 670 10.3390/polym11040670. 30979077 PMC6523900

[ref32] Rocca D. M.; Vanegas J. P.; Fournier K.; Becerra M. C.; Scaiano J. C.; Lanterna A. E. Biocompatibility and Photo-Induced Antibacterial Activity of Lignin-Stabilized Noble Metal Nanoparticles. RSC Adv 2018, 8 (70), 40454–40463. 10.1039/C8RA08169G. 35558201 PMC9091494

[ref33] Klapiszewski Ł.; Rzemieniecki T.; Krawczyk M.; Malina D.; Norman M.; Zdarta J.; Majchrzak I.; Dobrowolska A.; Czaczyk K.; Jesionowski T. Kraft Lignin/Silica–AgNPs as a Functional Material with Antibacterial Activity. Colloids Surf B Biointerfaces 2015, 134, 220–228. 10.1016/j.colsurfb.2015.06.056. 26204502

[ref34] Richter A. P.; Brown J. S.; Bharti B.; Wang A.; Gangwal S.; Houck K.; Cohen Hubal E. A.; Paunov V. N.; Stoyanov S. D.; Velev O. D. An Environmentally Benign Antimicrobial Nanoparticle Based on a Silver-Infused Lignin Core. Nat Nanotechnol 2015, 10 (9), 817–823. 10.1038/nnano.2015.141. 26167765

[ref35] Ma Y.; Dai J.; Wu L.; Fang G.; Guo Z. Enhanced Anti-Ultraviolet, Anti-Fouling and Anti-Bacterial Polyelectrolyte Membrane of Polystyrene Grafted with Trimethyl Quaternary Ammonium Salt Modified Lignin. Polymer (Guildf) 2017, 114, 113–121. 10.1016/j.polymer.2017.02.083.

[ref36] Jiao Y.; Niu L.; Ma S.; Li J.; Tay F. R.; Chen J. Quaternary Ammonium-Based Biomedical Materials: State-of-the-Art, Toxicological Aspects and Antimicrobial Resistance. Prog. Polym. Sci. 2017, 71, 53–90. 10.1016/j.progpolymsci.2017.03.001. 32287485 PMC7111226

[ref37] Denyer S. P.; Stewart G. S. A. B. Mechanisms of Action of Disinfectants. Int Biodeterior Biodegradation 1998, 41 (3–4), 261–268. 10.1016/S0964-8305(98)00023-7.

[ref38] Thorsteinsson T.; Másson M.; Kristinsson K. G.; Hjálmarsdóttir M. A.; Hilmarsson H.; Loftsson T. Soft Antimicrobial Agents: Synthesis and Activity of Labile Environmentally Friendly Long Chain Quaternary Ammonium Compounds. J. Med. Chem. 2003, 46 (19), 4173–4181. 10.1021/jm030829z. 12954069

[ref39] Kwaśniewska D.; Chen Y.-L.; Wieczorek D. Biological Activity of Quaternary Ammonium Salts and Their Derivatives. Pathogens 2020, 9 (6), 459 10.3390/pathogens9060459. 32531904 PMC7350379

[ref40] Kopecky F. Micellization and Other Associations of Amphiphilic Antimicrobial Quaternary Ammonium Salts in Aqueous Solutions. Pharmazie 1996, 51 (3), 135–144. 8900863

[ref41] Functional Textiles for Improved Performance, Protection and Health; Sun G., Ed.; Elsevier: Gang, 2011.

[ref42] Tiller J. C.; Liao C.-J.; Lewis K.; Klibanov A. M. Designing Surfaces That Kill Bacteria on Contact. Proceedings of the National Academy of Sciences 2001, 98 (11), 5981–5985. 10.1073/pnas.111143098. PMC33409 11353851

[ref43] Li K.; Bian S.; Zhen W.; Li H.; Zhao L. Performance, Crystallization and Rheological Behavior of Poly(Lactic Acid)/N-(2-Hydroxyl) Propyl-3-Trimethyl Ammonium Chitosan Chloride Intercalated Vermiculite Grafted Poly(Acrylamide) Nanocomposites. React Funct Polym 2021, 158, 104791 10.1016/j.reactfunctpolym.2020.104791.

[ref44] Nadagouda M. N.; Vijayasarathy P.; Sin A.; Nam H.; Khan S.; Parambath J. B. M.; Mohamed A. A.; Han C. Antimicrobial Activity of Quaternary Ammonium Salts: Structure-Activity Relationship. Medicinal Chemistry Research 2022, 31 (10), 1663–1678. 10.1007/s00044-022-02924-9.

[ref45] Buffet-Bataillon S.; Tattevin P.; Bonnaure-Mallet M.; Jolivet-Gougeon A. Emergence of Resistance to Antibacterial Agents: The Role of Quaternary Ammonium Compounds—a Critical Review. Int. J. Antimicrob. Agents 2012, 39 (5), 381–389. 10.1016/j.ijantimicag.2012.01.011. 22421329

[ref46] Culler M. D.; Bitman J.; Thompson M. J.; Robbins W. E.; Dutky S. R. Mastitis: I. In Vitro Antimicrobial Activity of Alkyl Amines Against Mastitic Bacteria. J Dairy Sci 1979, 62 (4), 584–595. 10.3168/jds.S0022-0302(79)83294-4. 379061

[ref47] Kapitanov I. V.; Jordan A.; Karpichev Y.; Spulak M.; Perez L.; Kellett A.; Kümmerer K.; Gathergood N. Synthesis, Self-Assembly, Bacterial and Fungal Toxicity, and Preliminary Biodegradation Studies of a Series of < scp > l</Scp> -Phenylalanine-Derived Surface-Active Ionic Liquids. Green Chem. 2019, 21 (7), 1777–1794. 10.1039/C9GC00030E.

[ref48] Kusumahastuti D. K. A.; Sihtmäe M.; Kapitanov I. V.; Karpichev Y.; Gathergood N.; Kahru A. Toxicity Profiling of 24 L-Phenylalanine Derived Ionic Liquids Based on Pyridinium, Imidazolium and Cholinium Cations and Varying Alkyl Chains Using Rapid Screening Vibrio Fischeri Bioassay. Ecotoxicol Environ Saf 2019, 172, 556–565. 10.1016/j.ecoenv.2018.12.076. 30776578

[ref49] Balgavý P.; Devínsky F. Cut-off Effects in Biological Activities of Surfactants. Adv. Colloid Interface Sci. 1996, 66, 23–63. 10.1016/0001-8686(96)00295-3. 8857708

[ref50] Boyce J. M. Quaternary Ammonium Disinfectants and Antiseptics: Tolerance, Resistance and Potential Impact on Antibiotic Resistance. Antimicrob Resist Infect Control 2023, 12 (1), 32 10.1186/s13756-023-01241-z. 37055844 PMC10099023

[ref51] Haldar J.; Kondaiah P.; Bhattacharya S. Synthesis and Antibacterial Properties of Novel Hydrolyzable Cationic Amphiphiles. Incorporation of Multiple Head Groups Leads to Impressive Antibacterial Activity. J. Med. Chem. 2005, 48 (11), 3823–3831. 10.1021/jm049106l. 15916434

[ref52] Zhang S.; Ding S.; Yu J.; Chen X.; Lei Q.; Fang W. Antibacterial Activity, *in Vitro* Cytotoxicity, and Cell Cycle Arrest of Gemini Quaternary Ammonium Surfactants. Langmuir 2015, 31 (44), 12161–12169. 10.1021/acs.langmuir.5b01430. 26474336

[ref53] An L.; Heo J. W.; Chen J.; Kim Y. S. Water-Soluble Lignin Quaternary Ammonium Salt for Electrospun Morphology-Controllable Antibacterial Polyvinyl Alcohol/ Lignin Quaternary Ammonium Salt Nanofibers. J Clean Prod 2022, 368, 133219 10.1016/j.jclepro.2022.133219.

[ref54] Chang L.; Duan W.; Huang S.; Chen A.; Li J.; Tang H.; Pan G.; Deng Y.; Zhao L.; Li D. Improved Antibacterial Activity of Hemp Fibre by Covalent Grafting of Quaternary Ammonium Groups. R. Soc. Open Sci. 2021, 8 (3), 201904 10.1098/rsos.201904. 33959349 PMC8074917

[ref55] Arnold W. A.; Blum A.; Branyan J.; Bruton T. A.; Carignan C. C.; Cortopassi G.; Datta S.; DeWitt J.; Doherty A.-C.; Halden R. U.; Harari H.; Hartmann E. M.; Hrubec T. C.; Iyer S.; Kwiatkowski C. F.; LaPier J.; Li D.; Li L.; Muñiz Ortiz J. G.; Salamova A.; Schettler T.; Seguin R. P.; Soehl A.; Sutton R.; Xu L.; Zheng G. Quaternary Ammonium Compounds: A Chemical Class of Emerging Concern. Environ. Sci. Technol. 2023, 57 (20), 7645–7665. 10.1021/acs.est.2c08244. 37157132 PMC10210541

[ref56] Liang Y.; Li H.; Ji J.; Wang J.; Ji Y. Self-Aggregation, Antimicrobial Activity and Cytotoxicity of Ester-Bonded Gemini Quaternary Ammonium Salts: The Role of the Spacer. Molecules 2023, 28 (14), 5469 10.3390/molecules28145469. 37513340 PMC10386392

[ref57] Acurio Cerda K.; Kathol M.; Purohit G.; Zamani E.; Morton M. D.; Khalimonchuk O.; Saha R.; Dishari S. K. Cationic Lignin as an Efficient and Biorenewable Antimicrobial Material. ACS Sustain Chem Eng 2023, 11 (28), 10364–10379. 10.1021/acssuschemeng.3c01414.

[ref58] Jiao G.-J.; Peng P.; Sun S.-L.; Geng Z.-C.; She D. Amination of Biorefinery Technical Lignin by Mannich Reaction for Preparing Highly Efficient Nitrogen Fertilizer. Int J Biol Macromol 2019, 127, 544–554. 10.1016/j.ijbiomac.2019.01.076. 30660565

[ref59] Mohan M. K.; Silenko O.; Krasnou I.; Volobujeva O.; Kulp M.; Ošeka M.; Lukk T.; Karpichev Y. Chloromethylation of Lignin as a Route to Functional Material with Catalytic Properties in Cross-Coupling and Click Reactions. ChemSusChem 2024, 17, e202301588 10.1002/cssc.202301588. 38279777

[ref60] Lignin: Historical, Biological, and Materials Perspectives, Copyright, Advisory Board, Foreword, Dedication; 1999; pp 742 i–viii. 10.1021/bk-2000-0742.fw001.

[ref61] Methods in Lignin Chemistry; Lin S. Y.; Dence C. W., Eds.; Springer Berlin Heidelberg: Berlin, Heidelberg, 1992.

[ref62] Characterization of Lignocellulosic Materials; Hu Q. T., Ed.; Wiley, 2008.

[ref63] Gellerstedt G.; Henriksson G. Lignins: Major Sources, Structure and Properties. In Monomers, Polymers and Composites from Renewable Resources; Elsevier, 2008; pp 201–224.

[ref64] Christensen B. T. Barley Straw Decomposition under Field Conditions: Effect of Placement and Initial Nitrogen Content on Weight Loss and Nitrogen Dynamics. Soil Biol Biochem 1986, 18 (5), 523–529. 10.1016/0038-0717(86)90010-6.

[ref65] Verrillo M.; Savy D.; Cangemi S.; Savarese C.; Cozzolino V.; Piccolo A. Valorization of Lignins from Energy Crops and Agro-industrial Byproducts as Antioxidant and Antibacterial Materials. J Sci Food Agric 2022, 102 (7), 2885–2892. 10.1002/jsfa.11629. 34755340

[ref66] Matos M.; Claro F. C.; Lima T. A. M.; Avelino F.; Hansel F. A.; Maciel G. M.; Lomonaco D.; Magalhães W. L. E. Acetone:Water Fractionation of Pyrolytic Lignin Improves Its Antioxidant and Antibacterial Activity. J Anal Appl Pyrolysis 2021, 156, 105175 10.1016/j.jaap.2021.105175.

[ref67] Daoud N. N.; Dickinson N. A.; Gilbert P. Antimicrobial Activity and Physico-Chemical Properties of Some Alkyldimethylbenzylammonium Chlorides. Microbios 1983, 37 (148), 73–85. 6413825

[ref68] Li F.; Weir M. D.; Xu H. H. K. Effects of Quaternary Ammonium Chain Length on Antibacterial Bonding Agents. J Dent Res 2013, 92 (10), 932–938. 10.1177/0022034513502053. 23958761 PMC3775374

[ref69] Zhang H.; Zhao S.; Li A.; Bian K.; Shen S.; Tao M.; Shi P. Structure-Dependent Antimicrobial Mechanism of Quaternary Ammonium Resins and a Novel Synthesis of Highly Efficient Antimicrobial Resin. Science of The Total Environment 2021, 768, 144450 10.1016/j.scitotenv.2020.144450. 33453537

[ref70] Gilbert P.; Al-taae A. Antimicrobial Activity of Some Alkyltrimethylammonium Bromides. Lett Appl Microbiol 1985, 1 (6), 101–104. 10.1111/j.1472-765X.1985.tb01498.x.

[ref71] Baudrion F.; Périchaud A.; Vacelet E. Influence of Concentration and Structure of Quaternary Ammonium Salts on Their Antifouling Efficiency Tested against a Community of Marine Bacteria. Biofouling 2000, 14 (4), 317–331. 10.1080/08927010009378424.

[ref72] Gilbert P.; Moore L. E. Cationic Antiseptics: Diversity of Action under a Common Epithet. J. Appl. Microbiol. 2005, 99 (4), 703–715. 10.1111/j.1365-2672.2005.02664.x. 16162221

[ref73] Tomlinson E.; Brown M. R. W.; Davis S. S. Effect of Colloidal Association on the Measured Activity of Alkylbenzyldimethylammonium Chlorides against Pseudomonas Aeruginosa. J. Med. Chem. 1977, 20 (10), 1277–1282. 10.1021/jm00220a010. 409841

[ref74] Jõul P.; Ho T. T.; Kallavus U.; Konist A.; Leiman K.; Salm O.-S.; Kulp M.; Koel M.; Lukk T. Characterization of Organosolv Lignins and Their Application in the Preparation of Aerogels. Materials 2022, 15 (8), 2861 10.3390/ma15082861. 35454554 PMC9029481

[ref75] Pupart H.; Jõul P.; Bramanis M. I.; Lukk T. Characterization of the Ensemble of Lignin-Remodeling DyP-Type Peroxidases from Streptomyces Coelicolor A3(2). Energies (Basel) 2023, 16 (3), 1557 10.3390/en16031557.

[ref76] Parm Ü.; Metsvaht T.; Sepp E.; Ilmoja M.-L.; Pisarev H.; Pauskar M.; Lutsar I. Impact of Empiric Antibiotic Regimen on Bowel Colonization in Neonates with Suspected Early Onset Sepsis. European Journal of Clinical Microbiology & Infectious Diseases 2010, 29 (7), 807–816. 10.1007/s10096-010-0931-1. 20446013

