# Supplementary material for: Synthesis and Antibacterial Properties of Novel Quaternary Ammonium Lignins
Source: ACS Omega. 2024 Sep 2;9(37):39134–45. doi: 10.1021/acsomega.4c06000 (PMC11411688; doi:10.1021/acsomega.4c06000)
Supplement: Supplementary file 1 — ao4c06000_si_001.pdf [file ao4c06000_si_001.pdf]

ELECTRONIC SUPPORTING INFORMATION

Synthesis and Antibacterial Properties of Novel Quaternary Ammonium Lignins

Mahendra K. Mohan,[†a] *Harleen Kaur,*[†b] *Merilin Rosenberg,*[b] *Ella Duvanova,*[a,c] *Tiit Lukk,*[a] *Angela Ivask,*[*b] *Yevgen Karpichev,* [*a]

[a] Department of Chemistry and Biotechnology, Tallinn University of Technology (TalTech), 15 Akadeemia Rd., 12618 Tallinn, Estonia

[b] Institute of Molecular and Cell Biology, University of Tartu, 23 Riia St., 51010 Tartu, Estonia

[c] Vasyl' Stus Donetsk National University, 21 600-richchia Vul., 21027 Vinnytsia, Ukraine

[†] *contributed equally to the publication*

Corresponding Author:

[*]Dr. Angela Ivask, phone: +372 737 5020

E-mail: angela.ivask@ut.ee

[*]Dr. Yevgen Karpichev, fax: +372 620 2994

E-mail: yevgen.karpichev@taltech.ee

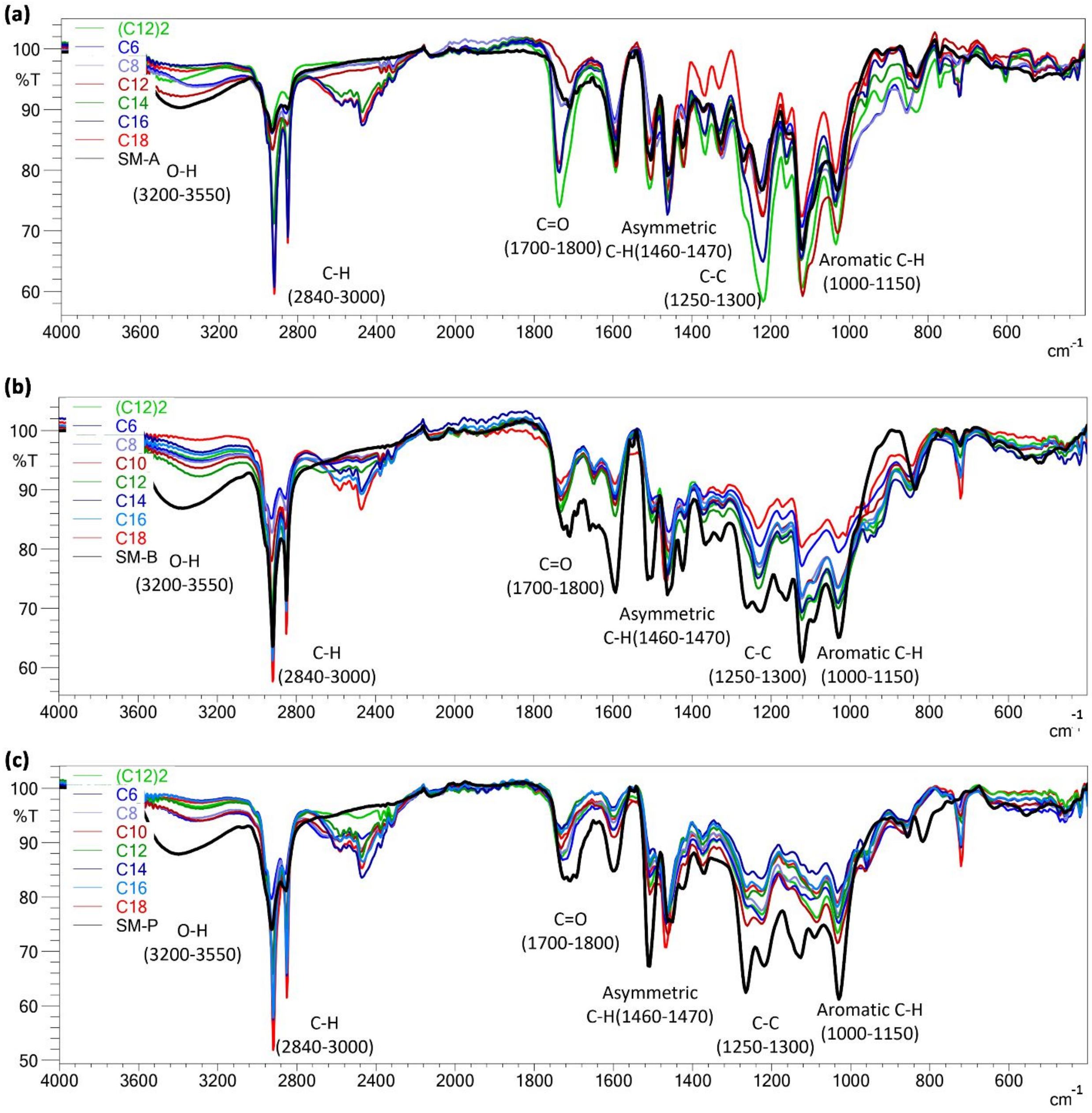

Figure S1. FT-IR spectra of quaternary ammonium lignins (QAL) of aspen (a), barley (b) and pine (c). For comparison, the spectra of organosolv lignins (SM) is shown as black bold line. Characteristic peaks are indicated.

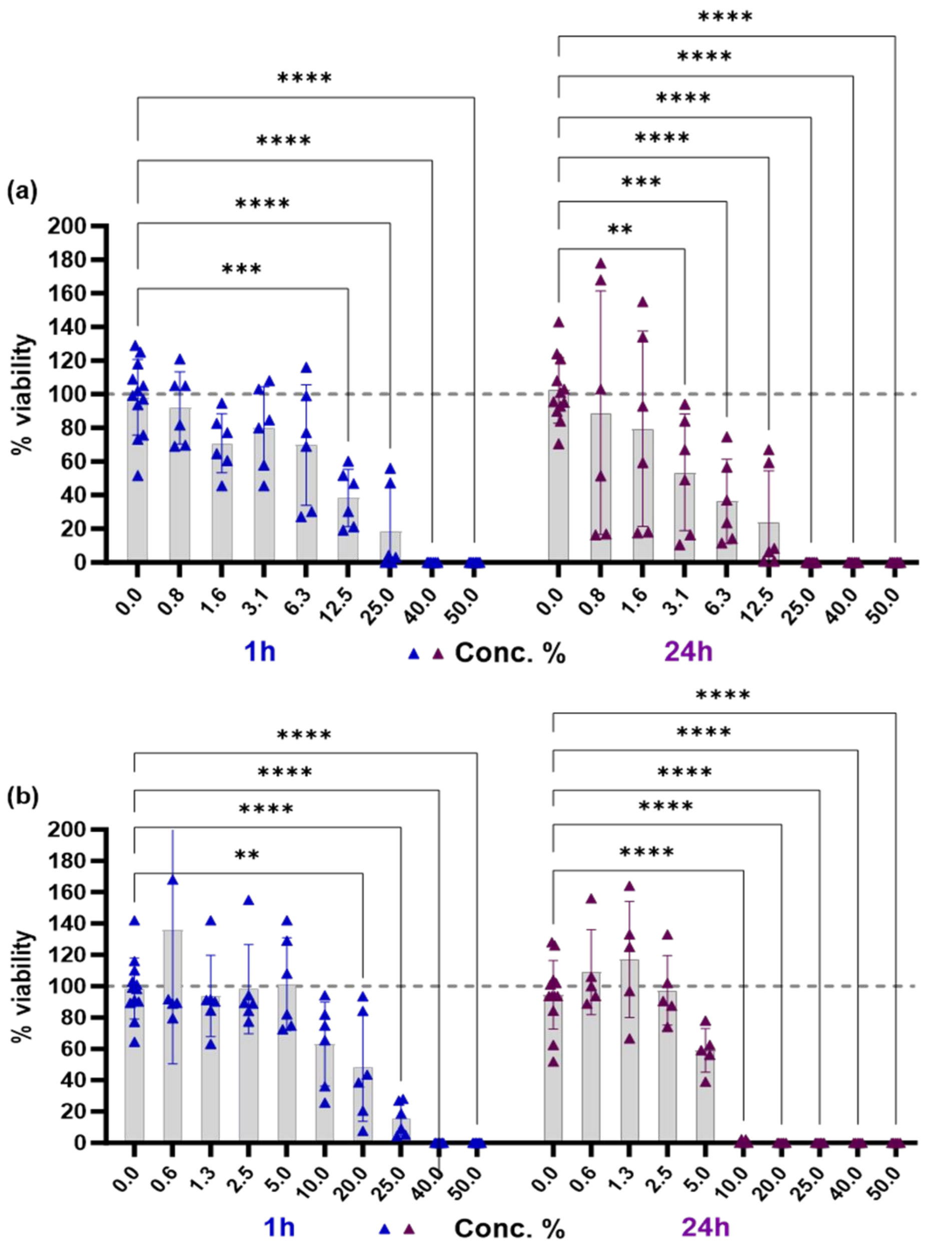

Figure S2. The effect of DMSO on bacterial viability. Viability was calculated based on (a) *Staphylococcus aureus* or (b) *Klebsiella pneumoniae* colony counts after 1 and 24 h exposure to different DMSO concentrations compared with 0% DMSO condition. Statistically significant differences from 0% DMSO control are shown as **** ($p \leq 0.0001$), *** ($p \leq 0.001$), and ** ($p \leq 0.01$). According to the most sensitive bacterium and timepoint (*Klebsiella pneumoniae* at 24 h of exposure), DMSO concentration of 1.5% that did not cause any significant decrease in bacterial viability was chosen for all MBC experiments with lignins

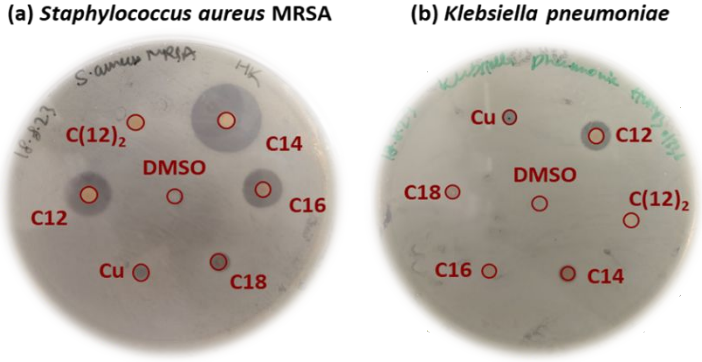

Figure S3. Zone of inhibition (ZOI) in growth inhibition test on agar for DMSO and quaternary ammonium lignins (QAL) for *S. aureus* and *K. pneumoniae*. A spot of DMSO is shown in the centre of the plates marked as the red circle, selected undiluted QALs (C12, C(12)$_2$, C14, C16, C18, 5% $CuSO_4$ (test control)) are shown in the outer circle. DMSO did not inhibit growth in the test format used.

Table S1. Minimal biocidal concentrations (MBC) of lignin and QAL samples against *S. aureus*. MBCs are expressed in mg/ml and shown for 1 h and 24 h exposure. Results for SM – organosolv lignin, CM – chloromethylated lignin, C6-C18, $C(12)_2$ – QALs from three experiments are shown.

MBC (mg/ml)							
**Aspen 1h**				**Aspen 24h**			
**SM**	>1.5	>1.5	>1.5	**SM**	0.19	0.19	0.19
**CM**	>1.5	>1.5	>1.5	**CM**	0.047	0.047	0.023
**C6**	>1.5	>1.5	>1.5	**C6**	0.023	0.023	0.023
**C8**	0.19	0.19	0.19	**C8**	0.023	0.012	0.023
**C12**	0.094	0.38	0.38	**C12**	0.023	0.012	0.012
**C14**	0.75	0.75	1.5	**C14**	0.047	0.047	0.023
**C16**	0.094	0.094	0.19	**C16**	0.023	0.023	0.012
**C18**	0.047	0.047	0.094	**C18**	0.023	0.023	0.012
**$C(12)_2$**	0.38	0.38	0.38	**$C(12)_2$**	0.023	0.012	0.012
**Barley 1h**				**Barley 24h**			
**SM**	>1.5	>1.5	>1.5	**SM**	0.38	0.19	0.19
**CM**	>1.5	>1.5	>1.5	**CM**	0.094	0.047	0.094
**C6**	>1.5	>1.5	>1.5	**C6**	0.38	0.38	0.38
**C8**	>1.5	>1.5	>1.5	**C8**	0.75	0.38	0.75
**C10**	>1.5	>1.5	>1.5	**C10**	0.75	0.75	0.75
**C12**	>1.5	>1.5	>1.5	**C12**	0.38	0.38	0.19
**C14**	0.19	0.38	0.75	**C14**	0.023	0.023	0.012
**C16**	0.094	0.094	0.38	**C16**	0.023	0.023	0.012
**C18**	0.047	0.047	0.047	**C18**	0.012	0.012	0.012
**$C(12)_2$**	0.38	0.38	0.38	**$C(12)_2$**	0.047	0.023	0.012
**Pine 1h**				**Pine 24h**			
**SM**	1.5	>1.5	>1.5	**SM**	0.012	0.094	0.094
**CM**	1.5	>1.5	1.5	**CM**	0.047	0.023	0.047
**C6**	>1.5	>1.5	>1.5	**C6**	0.19	0.38	0.19
**C8**	0.38	>1.5	1.5	**C8**	0.19	0.19	0.19
**C10**	0.19	0.38	0.38	**C10**	0.094	0.047	0.094
**C12**	0.38	0.75	0.38	**C12**	0.047	0.023	0.023
**C14**	0.38	0.38	0.75	**C14**	0.047	0.023	0.012
**C16**	0.023	0.094	0.094	**C16**	0.012	0.012	0.012
**C18**	0.023	0.023	0.023	**C18**	0.012	0.012	0.0059
**$C(12)_2$**	0.38	0.38	0.38	**$C(12)_2$**	0.023	0.023	0.012

Table S2. Minimal biocidal concentrations (MBC) of lignin and QAL samples against *K. pneumoniae*. MBCs are expressed in mg/ml and shown for 1 h and 24 h exposure. Results for SM - organosolv lignin, CM - chloromethylated lignin, C6-C18, $C(12)_2$ - QALs from three experiments are shown.

MBC (mg/ml)							
	Aspen 1h				Aspen 24h		
SM	>1.5	>1.5	>1.5	SM	>1.5	1.5	1.5
CM	>1.5	1.5	1.5	CM	0.38	0.19	0.19
C6	0.023	0.023	0.023	C6	0.023	0.023	0.023
C8	0.023	0.023	0.012	C8	0.023	0.023	0.023
C12	0.012	0.012	0.012	C12	0.023	0.047	0.012
C14	0.094	0.047	0.094	C14	0.023	0.012	0.012
C16	0.023	0.023	0.023	C16	0.023	0.012	0.012
C18	0.023	0.047	0.023	C18	0.023	0.012	0.012
$C(12)_2$	0.047	0.012	0.012	$C(12)_2$	0.023	0.012	0.012
	Barley 1h				Barley 24h		
SM	>1.5	>1.5	>1.5	SM	>1.5	>1.5	>1.5
CM	>1.5	0.75	1.5	CM	0.38	0.19	0.19
C6	>1.5	1.5	1.5	C6	0.38	0.38	0.38
C8	>1.5	1.5	0.75	C8	0.38	0.19	0.19
C10	>1.5	>1.5	>1.5	C10	0.75	0.19	0.38
C12	0.75	0.75	0.75	C12	0.047	0.047	0.047
C14	0.023	0.023	0.023	C14	0.012	0.0059	0.0059
C16	0.023	0.023	0.023	C16	0.012	0.012	0.012
C18	0.023	0.023	0.023	C18	0.023	0.012	0.023
$C(12)_2$	0.19	0.094	0.094	$C(12)_2$	0.023	0.012	0.012
	Pine 1h				Pine 24h		
SM	>1.5	>1.5	>1.5	SM	>1.5	>1.5	>1.5
CM	>1.5	>1.5	>1.5	CM	0.38	0.19	0.19
C6	0.75	0.38	0.094	C6	0.094	0.094	0.094
C8	0.19	0.19	0.094	C8	0.094	0.094	0.19
C10	0.094	0.047	0.047	C10	0.047	0.047	0.0478
C12	0.047	0.047	0.047	C12	0.023	0.023	0.0234
C14	0.047	0.023	0.023	C14	0.012	0.012	0.012
C16	0.012	0.012	0.012	C16	0.012	0.012	0.0059
C18	0.012	0.023	0.012	C18	0.012	0.0059	0.012
$C(12)_2$	0.19	0.094	0.19	$C(12)_2$	0.012	0.012	0.012

Table S3. Zone of inhibition (ZOI, mm) for *S. aureus* and *K. pneumoniae* measured in growth inhibition test around the spots of different lignins and QALs after 24 h incubation.

	Staphylococcus aureus, ZOI (mm)								
	Aspen			Barley			Pine		
**SM**	0	0	0	0	0	0	0	0	0
**CM**	0	0	0	0	0	0	0	0	0
**C6**	2.2	2.0	2.2	0	0	0	0.7	0.9	1.1
**C8**	2.8	3.4	3.3	0	0.3	0.2	1.0	1.2	1.2
**C10**	n.a.	n.a.	n.a.	0.7	0.5	0.6	0.9	1.2	1.3
**C12**	2.1	2.6	3.1	1.9	2.6	3.5	3.7	2.3	5.0
**C14**	4.7	5.1	4.4	6.2	6.2	6.5	4.9	5.8	4.8
**C16**	3.3	3.1	2.7	2.8	3.2	2.9	3.0	3.7	3.0
**C18**	1.1	1.0	1.1	0.9	1.1	1.1	0.7	1.2	1.2
**$C(12)_2$**	1.3	1.6	1.6	0	0	0	0	0	0
	Klebsiella pneumoniae, ZOI (mm)								
	Aspen			Barley			Pine		
**SM**	0	0	0	0	0	0	0	0	0
**CM**	0	0	0	0	0	0	0	0	0
**C6**	0	0	0	0	0	0	0	0	0
**C8**	0	0	0	0.2	0.4	0.3	0	0	0
**C10**	0.7	0.6	0.5	0.7	0.5	0.4	0.5	0.6	0.7
**C12**	1.3	1.6	1.9	1.3	0.9	1.7	2.5	2.3	2.7
**C14**	0.3	0.4	0.5	0.8	0.5	1	0.9	0.3	0.8
**C16**	0	0	0	0	0	0	0	0	0
**C18**	0	0	0	0	0	0	0	0	0
**$C(12)_2$**	0	0	0	0	0	0	0	0	0
